# The global distribution of Crimean-Congo hemorrhagic fever

**DOI:** 10.1093/trstmh/trv050

**Published:** 2015-07-04

**Authors:** Jane P. Messina, David M. Pigott, Nick Golding, Kirsten A. Duda, John S. Brownstein, Daniel J. Weiss, Harry Gibson, Timothy P. Robinson, Marius Gilbert, G. R. William Wint, Patricia A. Nuttall, Peter W. Gething, Monica F. Myers, Dylan B. George, Simon I. Hay

**Affiliations:** a Department of Zoology, University of Oxford, Oxford, UK; b Wellcome Trust Centre for Human Genetics, University of Oxford, Oxford, UK; c Department of Pediatrics, Harvard Medical School and Children's Hospital Informatics Program, Boston Children's Hospital, Boston, MA, USA; d Livestock Systems and Environment (LSE), International Livestock Research Institute (ILRI),Nairobi, Kenya; e Biological Control and Spatial Ecology, Université Libre de Bruxelles, Brussels, Belgium; f Fonds National de la Recherche Scientifique, Brussels, Belgium; g Fogarty International Center, National Institutes of Health, Bethesda, MD, USA; h Institute for Health Metrics and Evaluation, University of Washington, Seattle, WA, USA

**Keywords:** Crimean-Congo hemorrhagic fever, Crimean-Congo hemorrhagic fever virus, Ecological niche modeling, Infectious diseases, Tick-borne diseases, Vector-borne diseases

## Abstract

**Background:**

Crimean-Congo hemorrhagic fever (CCHF) is a tick-borne infection caused by a virus (CCHFV) from the Bunyaviridae family. Domestic and wild vertebrates are asymptomatic reservoirs for the virus, putting animal handlers, slaughter-house workers and agricultural labourers at highest risk in endemic areas, with secondary transmission possible through contact with infected blood and other bodily fluids. Human infection is characterized by severe symptoms that often result in death. While it is known that CCHFV transmission is limited to Africa, Asia and Europe, definitive global extents and risk patterns within these limits have not been well described.

**Methods:**

We used an exhaustive database of human CCHF occurrence records and a niche modeling framework to map the global distribution of risk for human CCHF occurrence.

**Results:**

A greater proportion of shrub or grass land cover was the most important contributor to our model, which predicts highest levels of risk around the Black Sea, Turkey, and some parts of central Asia. Sub-Saharan Africa shows more focalized areas of risk throughout the Sahel and the Cape region.

**Conclusions:**

These new risk maps provide a valuable starting point for understanding the zoonotic niche of CCHF, its extent and the risk it poses to humans.

## Introduction

Crimean-Congo hemorrhagic fever (CCHF) is a tick-borne viral (Nairovirus, family Bunyaviridae) infection first identified in the Crimean region in 1944.^1,2^ It was subsequently shown to be the same virus as that causing similar hemorrhagic disease outbreaks in the Congo basin, giving the virus its current name.^3,4^ CCHF is one of the most widely distributed arboviral diseases in the world, ranging from southern Russia and the Black Sea region to the southern tip of Africa.^4^ The disease is considered as ‘emerging’ across the globe, with many countries reporting new infections in humans in recent decades, including Albania (2001),^5^ Turkey (2002)^6^ and Georgia (2009).^7^ In some regions, human CCHF infection has also been recently reported after long periods of absence, for example in south-western Russia^8^ and Central Africa.^9^

**Figure 1.**
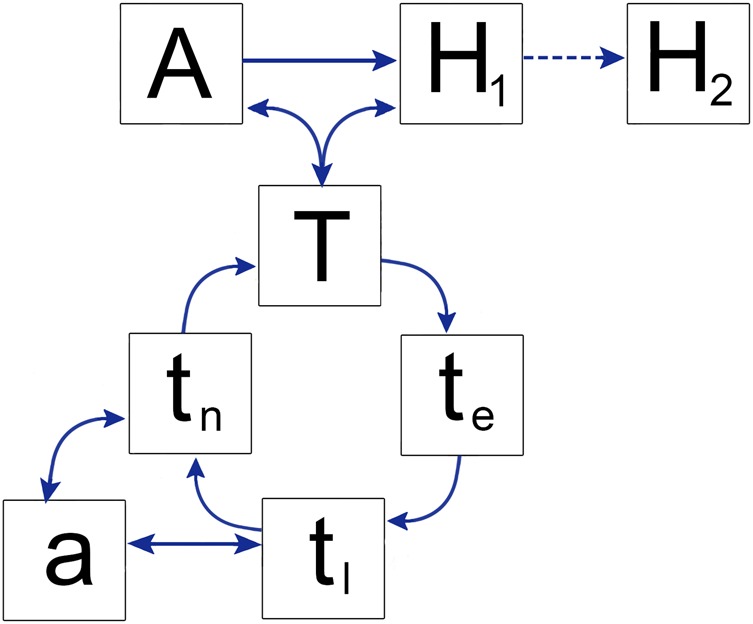
Transmission cycle of Crimean-Congo hemorrhagic fever virus (CCHFV) where t_e,_ t_l_, and t_n_ represent the eggs, larvae, and nymphs of competent tick vectors, respectively. Nymphs (t_n_) transmit CCHFV to small mammals and birds (a), whereas transmission to ruminants and other large animals (A) is by adult ticks (T). Primary human infections (H_1_) occur as a result of being directly bitten by adult ticks or squashing ticks between the fingers (T), or through contact with the blood of infected animals, usually livestock (A). The comparatively rarer human-to-human transmission (represented by the dashed line from H_1_ to H_2_) is typically between infected individuals and healthcare workers or close relatives having exposed to their infectious blood and/or bodily fluids.^88^

While no apparent disease manifestation occurs in animals,^10^ both wild and domesticated animals represent an important link in the disease transmission cycle, acting as reservoirs for continued tick re-infection (Figure 1). Many tick species have been associated with CCHF virus (CCHFV), but members of the genus *Hyalomma* are considered the primary vectors and are the most common ticks known to transmit the virus to humans.^11^ These ticks are adapted to hot and dry or semiarid environments, and are found in many parts of Africa, Asia, and Europe.^1,12–15^

Infection of humans is a comparatively rare event, with those living or working in close proximity to livestock (particularly cattle, sheep and goats) or tick vector habitats being particularly at risk of infectious tick bites, and those working in animal slaughterhouses being at risk for blood-borne exposure.^16,17^ Human-to-human transmission is possible, typically amongst healthcare workers or close relatives having close contact and exposure to infectious blood or bodily fluids of those infected with CCHFV.^16,18,19^ There is no widely available safe and effective vaccine against CCHF, although a recombinant CCHFV vaccine candidate has shown good in vivo efficacy.^20^ Currently, treatment for the potentially fatal disease remains largely supportive.^21,22^ Personal protective measures such as the use of pyrethroid acaricides and wearing protective clothing are important, but generally there is little knowledge about these measures in areas where current levels of risk are ill-defined.^11,23^

Currently, spatial analyses of CCHF are few in number compared to those for many other diseases and even other tick-borne viruses.^24,25^ Still, several studies have elucidated the chief drivers of CCHF geographic distribution patterns. Strong correlations have been found in Turkey and Bulgaria between CCHF risk and suitable environments for *Hyalomma* ticks, including grass and shrub cover, as well as forested land fragmented by agricultural or shrub cover.^26–29^ Non-irrigated agricultural land cover (e.g., pasture and rangeland) has also been found to be associated with CCHF incidence in Turkey and Greece.^26,27,30^ The CCHFV infection rate in livestock was found to be a strong positive predictor of CCHF incidence in humans in Iran and Mauritania,^31,32^ although in Bulgaria where vaccination coverage is high amongst at-risk populations (e.g., veterinarians and farm workers), livestock density was not a significant driver of CCHF incidence in humans.^29^ Climate indicators have also been found as important predictors of CCHF risk. Areas regularly experiencing long periods of low rainfall and humidity were associated with increased occurrence of CCHF in Iran and Senegal,^33,34^ and higher temperatures were indicators of CCHF occurrence in Turkey, Bulgaria, and Iran.^28,29,33^

Existing global distribution maps of CCHF are largely in the form of national-level maps of vector presence or reported human cases, such as that provided by WHO.^35^ Here we draw upon the findings of several of the country-specific studies to model risk for CCHF infection in humans at a global scale using an ecological niche modeling approach. This approach enables us to better identify at-risk areas by using environmental correlations found in areas of good CCHF reporting to predict risk in those areas where less is known about transmission of the disease. While a preliminary CCHF risk map was produced using a similar statistical approach in the past,^36^ the current paper offers a more recent and refined global geographic estimation of the distribution of CCHF. This was made possible by the addition of new data for the locations of disease occurrence, an evidence-based consensus layer for background data sampling, and high-resolution environmental layers alongside newer methodologies. Because the geographic distribution of CCHF is taken into account when patients' travel histories are considered during differential diagnosis of hemorrhagic fevers,^37^ an up-to-date and high-resolution map of the global distribution of the disease is essential. As the maps we provide define regions not only where CCHF has been reported but also where transmission is possible, successful identification of both locally acquired and imported cases^38–40^ may be expedited, therefore reducing the likelihood of further secondary human-to-human transmission. The recent Ebola virus outbreak in West Africa has highlighted the critical nature of such considerations.^41^ In several African countries, the risk of CCHF is poorly defined meaning infection with CCHFV is more likely to go undiagnosed or unreported in this region. An improved understanding of the geographic extents of CCHF and the true level of risk within these extents is vital for increasing awareness about the disease, advocating for improved individual protection from *Hyalomma* tick bites, and promoting safe practices for slaughterhouse and healthcare workers. Finally, this work contributes to a wider initiative to better map the ecological niche of several of the viral hemorrhagic fevers occurring in Africa which not only pose the risk of zoonotic transmission, but also of secondary nosocomial and community-level transmission.^37,42,43^

## Methods

**Figure 2.**
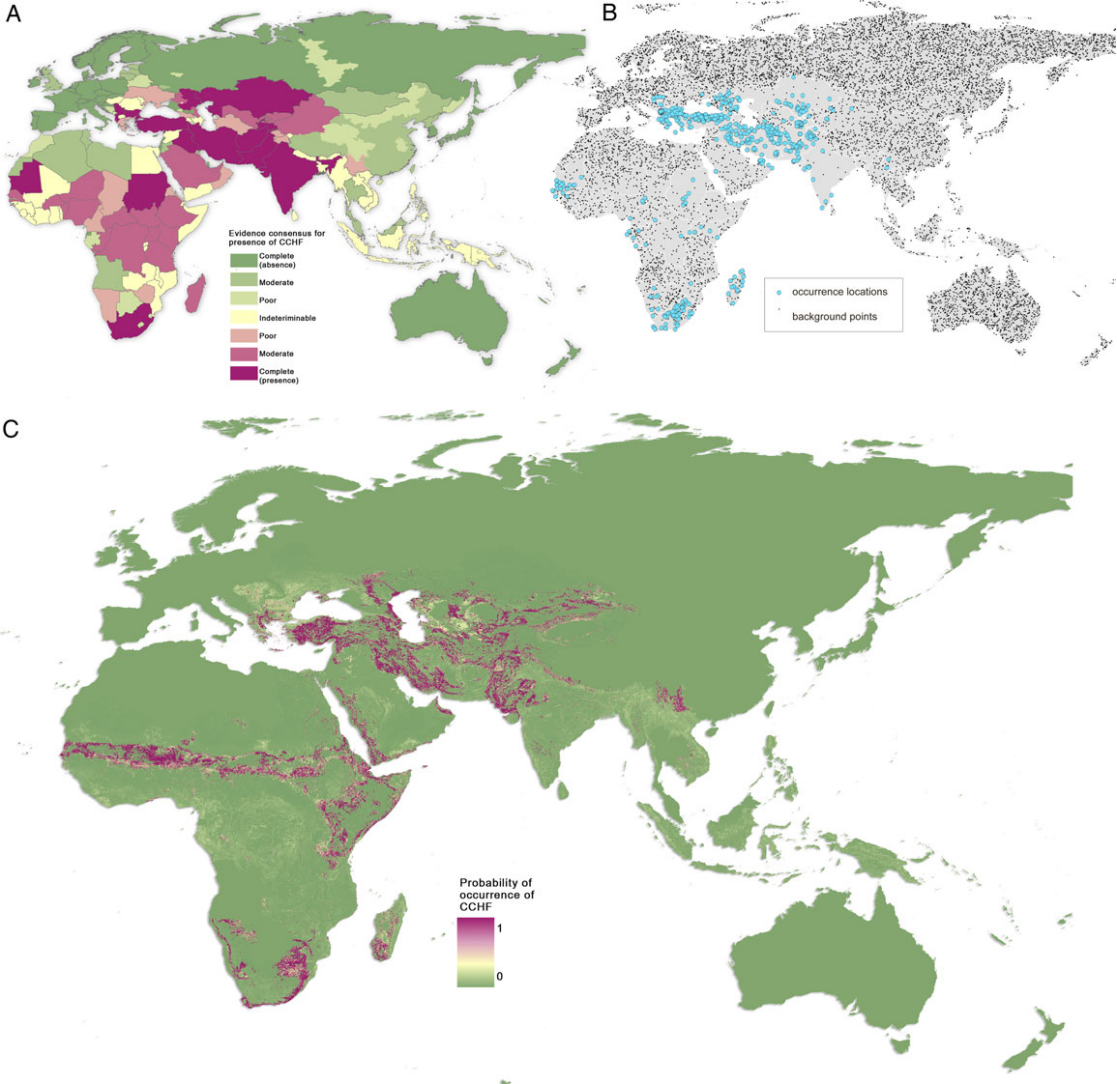
Maps of A: definitive extents as determined by evidence consensus; B: recorded occurrence and generated background points used in the BRT procedure; and C: probability of occurrence of Crimean-Congo haemorrhagic fever (CCHF). A: shows the consensus on CCHF presence globally, ranging from dark green (complete consensus on absence) to purple (complete consensus on presence). Countries in yellow are those where evidence was inconclusive or contradictory for CCHF presence. B: shows the probability of CCHF occurrence in humans. Areas in purple are those most suitable for transmission, with areas in green least suitable.

We used boosted regression trees (BRT), a method for modeling species distributions, to create maps of environmental suitability for CCHF occurrence. Our particular approach has been successfully employed in similar disease mapping efforts,^44,45^ and requires the generation of: a layer assessing the strength of evidence for CCHF presence or absence, termed evidence consensus, at a national and sometimes sub-national level^46^ (Figure 2A); a comprehensive database of the locations of CCHF reports in humans (Figure 2B); and a suite of globally gridded environmental and socioeconomic covariates known or hypothesised to affect CCHF transmission. The output map presents a probabilistic surface of environmental suitability of CCHF occurrence (‘CCHF risk’) within its global geographic extents at a 5 km×5 km spatial resolution (Figure 2C).

### Definitive extents

We carried out a process consisting of four components (described below) to evaluate the certainty of presence or absence of CCHF for each country and certain select sub-national regions at the edges of its distribution. The methodology used to generate the definitive extents of CCHF was adapted from that used for dengue and is termed evidence consensus.^45,46^ The information used to determine the final score for each country or sub-national region is provided in Supplementary Table 1.

#### Health reporting organization evidence (max ±3)

Evidence from WHO^35^ and the Global Infectious Diseases and Epidemiology Online Network (GIDEON)^47^ was used. WHO places each nation into one of five categories of evidence for CCHF: absent; *Hyalomma* ticks present; CCHF virological or serological evidence and vector present; 5–49 CCHF cases reported per year; and 50 or more CCHF cases reported per year. GIDEON listed each country as either endemic or non-endemic; if the country was not listed, the entry was recorded as unspecified. Quantitative scores for each unique permutation are laid out in Table 1.

**Table 1.** Derivation of quantitative scores for health-reporting organization evidence

GIDEON	WHO	Score
Endemic	50+ CCHF cases reported per year	+3
	5–49 CCHF cases reported per year	+2.5
	CCHF virological or serological evidence and vector present	+2
	*Hyalomma* tick vector present	+1.5
	Absent	0
Unspecified	50+ CCHF cases reported per year	+2
	5–49 CCHF cases reported per year	+1.5
	CCHF virological or serological evidence and vector present	+1
	*Hyalomma* tick vector present	+0.5
	Absent	−2
Non-endemic	50+ CCHF cases reported per year	−0.5
	5–49 CCHF cases reported per year	−1
	CCHF virological or serological evidence and vector present	−1.5
	*Hyalomma* tick vector present	−2
	Absent	−3

CCHF: Crimean-Congo hemorrhagic fever; GIDEON: Global Infectious Diseases and Epidemiology Online Network.

#### Peer-reviewed evidence (max +6)

We conducted a country-specific search on PubMed and Web of Science using the terms ‘[country] CCHF’ or ‘[country] Crimean Congo Hemorrhagic Fever’ or ‘[country] Crimean Hemorrhagic Fever’ or ‘[country] Congo Hemorrhagic Fever’. Reported cases in the literature were evaluated based upon their contemporariness and diagnostic accuracy. Contemporariness was evaluated in three categories: 2006–2013=3, 1998–2005=2, 1997 and earlier=1. Different diagnostic techniques were scored in a similar banding system, with PCR techniques, or genotyping achieving 3 points, 2 awarded for the use of IgM- and IgG-based ELISA or other serological techniques, and with 1 point for cases that were just reported or referred to an unspecified ‘laboratory diagnosis’.

#### Case data (max ±6)

Data on reported outbreaks of CCHF, with a threshold of five cases, were obtained from GIDEON datasets and the literature. Outbreaks above this threshold were scored for their contemporariness with outbreaks before 1998=+2, 1998–2005=+4, 2006–2013=+6. If there were no reported outbreaks, healthcare expenditure as reported by the WHO was used as a proxy for diagnostic capacity in an attempt to differentiate genuine absence or sporadic cases from inability to adequately diagnose CCHF. Expenditure was stratified annually per capita at average US$ exchanges rates (2011 US$ WHO Health Statistics) with three categories defined: HE Low (<US$100), HE Medium (US$100 to <US$500) and HE High (>US$500). These measures are used as a proxy for the quality of healthcare reporting, as it is unlikely that vast numbers of CCHF infections have gone undetected in countries where healthcare expenditure is high (e.g., in northern European countries).

#### Supplementary evidence (max ±3)

In cases where contradictory evidence led to uncertainty in the presence or absence of CCHF, supplementary evidence was supplied. Here, seroepidemiological surveys, or the presence of CCHFV in ticks or livestock were evaluated and scored. Country-specific scoring is outlined in Supplementary Table 1 where appropriate.

### Assembly of the occurrence database

An occurrence database comprising point (e.g., town or city) or polygon (e.g., county or province) locations of confirmed CCHF infection presence was compiled from peer-reviewed literature, Genbank records, and HealthMap alerts.^48,49^ A literature search was conducted on PubMed and Web of Science using the terms ‘CCHF’ or ‘Crimean Congo Hemorrhagic Fever’ or ‘Crimean Hemorrhagic Fever’ or ‘Congo Hemorrhagic Fever’. The same terms were used in our Genbank search. An occurrence was defined as one or more laboratory or clinically confirmed infection(s) of CCHF occurring at a unique location (the same administrative area or 5 km×5 km pixel for points) within one calendar year. All occurrence data underwent manual review and quality control to ensure information fidelity and precise geo-positioning. Reports of autochthonous (locally transmitted) cases or outbreaks were entered as an occurrence within the country in which transmission occurred. If imported cases were reported with information about the site of infection, they were geo-positioned to the country where transmission occurred. If imported cases were reported with no information about the site of contagion, they were not entered into the database. In addition, polygons greater than one square degree in area at the equator were removed from the database, as their inclusion in niche modeling would introduce a large amount of bias. This database has been made publicly available for download.^50^

### Explanatory covariates

We assembled gridded global data (5 km×5 km) for a set of five explanatory covariates. The covariates were chosen based on factors known or hypothesised to contribute to suitability for CCHF transmission based upon the national-level studies described in the introduction. These included annual mean precipitation interpolated from global meteorological stations^51^ and mean land surface temperature derived from NASA's moderate resolution imaging spectrometer (MODIS) imagery,^52^ intended to capture the generally warm and arid climate zones where CCHFV is transmitted. We also included a 5 km×5 km resolution measure of the mean annual Enhanced Vegetation Index (EVI; also from MODIS)^53^ (computed from the original 1 km×1 km data set), as well as the SD of this mean, which is intended to serve as a proxy for landscape diversity and habitat fragmentation. All these surfaces were parsed through a gap-filling algorithm prior to inclusion in the analysis.^54^ The proportion of each 5 km×5 km grid cell covered by shrub or grass land cover types was also derived using the MODIS MCD12Q1 dataset which was originally obtained at 1 km×1 km resolution. To do this, we computed the proportion of 1 km×1 km grid cells within the larger 5 km×5 km cells that were classified as either grass, open shrub or closed shrub. The International Geosphere-Biosphere Programme (IGBP) land cover classification scheme was utilized.^55^ No covariate grids were shown to be adversely affected by multicollinearity and were standardised to ensure identical spatial resolution, extent, and boundaries. Maps for each covariate are provided in Supplementary Figure 1.

### Modeling risk for CCHF occurrence

We used a boosted regression tree (BRT) approach to establish a multivariate empirical relationship between the probability of CCHF presence and the environmental conditions (as determined by the set of covariates described above) sampled at each occurrence location. This method combines regression trees^56^ with gradient boosting,^57^ whereby an initial regression tree is fitted and iteratively improved upon in a forward stagewise manner (boosting) by minimising the variation in the response not explained by the model at each iteration. It has been shown to fit complicated response functions efficiently, while guarding against over-fitting, and has thus been applied in the past for vector and disease distribution mapping.^42,44,45,58–61^

A large proportion of the point-level records in our occurrence database were geo-positioned to urban areas where cases are more likely to have been diagnosed rather than acquired (based on the ecology of CCHF tick vectors). Because of this, there is uncertainty about where in the vicinity CCHFV transmission actually took place. In an effort to reduce spatial bias, we thus assumed that when a location was recorded as a point, transmission could have taken place anywhere within the larger administrative corresponding to the FAO's Admin2-level Global Administrative Unit Layers (GAUL),^62^ which typically represents counties or municipalities. We then calculated the mean of all covariates within these polygons and for any records which were originally recorded as polygons. While this approach reduced bias toward urban areas in our models, it is limited in its assumption that Admin2 units are correct for sampling environmental correlates of CCHF and in its disregard for variation in covariate values within polygons.

Like other ecological niche-mapping approaches, the BRT models require not only presence data but also background data defining areas of potentially unsuitable environmental conditions at unsampled locations, since data on absence of disease are rarely reported. No consensus approach has been developed to optimise the generation of background data and we therefore created an evidence-based probabilistic framework for generating pseudo-data. To represent the environmental conditions in locations where the disease has not been reported, 10 000 background points^63–65^ were randomly generated and weighted based on a continuous raster surface derived from the national (and sometimes sub-national) CCHF evidence consensus scores. As such, more background points were located in areas with high consensus on absence.

To increase the robustness of model predictions and quantify model uncertainty, we fitted an ensemble^66^ of 100 BRT models to separate bootstraps of the data. We then evaluated the central tendency as the mean across all 100 BRT models (see Bhatt et al.^44^). Each of the 100 individual models was fitted using the gbm.step subroutine in the dismo package in the R statistical programming environment.^67^ All other tuning parameters of the algorithm were held at their default values (tree complexity=4, learning rate=0.005, bag fraction=0.75, step size=10, cross-validation folds=10). One 5 km×5 km pixel was randomly selected from within each polygon for each individual model in order to account for the environmental uncertainty associated with imprecise geographic data. In order to improve the weighting capacity of each of the 100 models, weightings were applied to the background dataset such that the sum of the weighted background data equalled the weighted sum of the occurrence records.^68^ Each of the 100 models predicts environmental suitability on a continuous scale from 0 to 1, with a final prediction map then being generated by calculating the mean prediction across all models for each 5 km×5 km pixel. Cross-validation was applied to each model, whereby 10 subsets of the data comprising 10% of the presence and background observations were assessed based on their ability to predict the distribution of the other 90% of records using the mean area under the curve (AUC) statistic. This AUC value was then averaged across the 10 sub-models and finally across all 100 models in the ensemble in order to derive an overall estimate of goodness-of-fit. Additionally, to avoid AUC inflation due to spatial sorting bias, a pairwise distance sampling procedure was used,^69^ resulting in a final AUC which is lower than would be returned by standard procedures but which gives a more realistic quantification of the model's ability to extrapolate predictions to new regions.^70^

## Results

In total, 1721 occurrence records were included in our final dataset after performing all quality control procedures. These included 1470 county or district-level occurrences and 251 province-level occurrences spanning 1953 to 2012. We assumed that any recorded location of CCHF occurrence, regardless of the date of the record, represented an environment permissible for the disease.

The evidence consensus map (Figure 2A) showed 47 countries to have an indeterminate status in terms of CCHF presence or absence (scores between −15 and +15), as well as certain parts of China and Russia. Those with particularly poor evidence (score of zero) are mostly located in sub-Saharan Africa, but also include Cambodia, Laos, Myanmar, Vietnam, Nepal and Arunachal Pradesh in Asia, as well as Azerbaijan, Bhutan, Yemen, Moldova and Macedonia in the region spanning eastern Europe to central Asia. More information is needed about the possible occurrence of CCHF in these places, as well as the presence of any ticks that have been proven as competent vectors of CCHFV. Yunnan province in China is classified as having some evidence for CCHF presence due to CCHFV seropositivity having been found in humans,^71^ although the overall evidence is weak since no cases of human disease have been reported in the province. According to our evidence consensus measure, the five countries currently having the strongest evidence for CCHF presence are Turkey, Iran, Afghanistan, Tajikistan, and Pakistan.

**Figure 3.**
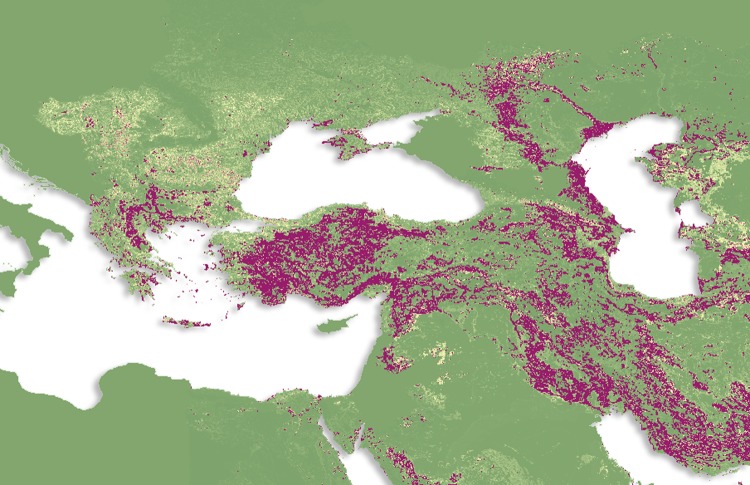
The probability of occurrence of Crimean-Congo haemorrhagic fever (CCHF) in the Balkans region. Areas in purple are those most suitable for transmission, with areas in green least suitable.

**Figure 4.**
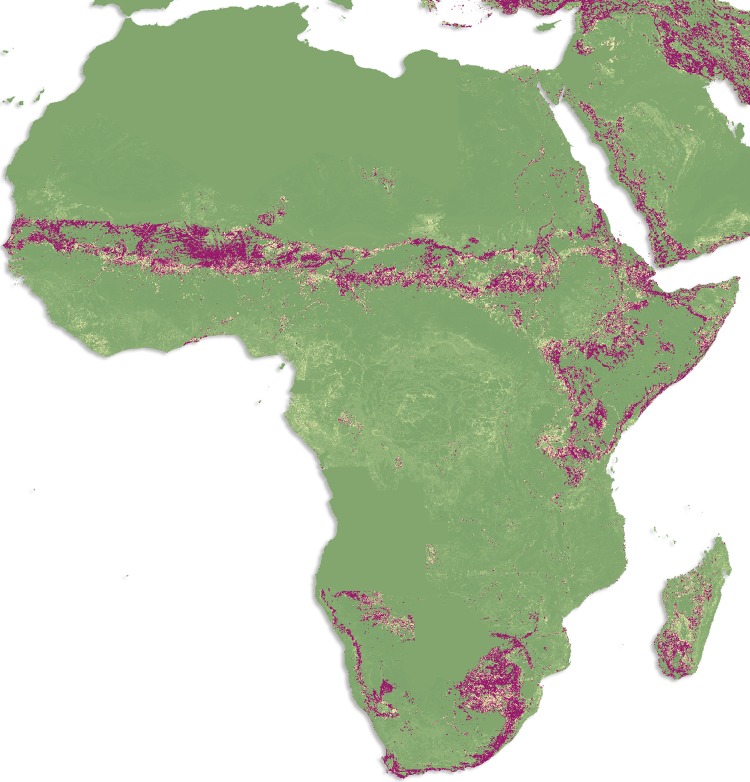
Probability of occurrence of Crimean-Congo haemorrhagic fever in Africa. Areas in purple are those most suitable for transmission, with areas in green least suitable.

The average of the ensemble of BRT models predicted high levels of risk for CCHF in the Black Sea region and some parts of central Asia, with more focalized areas of risk being found in the Sahel and Cape regions of Africa (Figure 2C). The countries with the largest areas of high risk for CCHF occurrence are Turkey, Iran, Romania, Moldova and Ukraine, with some parts of southwest Russia, Syria, Iraq and central Asia demonstrating high probabilities of occurrence as well (Figure 3). Although the evidence consensus for CCHF presence is strong for many countries in sub-Saharan Africa, our model predicts that areas with the highest probability of occurrence are overall much smaller in area and more irregularly distributed in this region than in the Black Sea region (see Figure 4). However, this may be an artefact of the small number of occurrence observations available for CCHF in Africa, as can be seen in Figure 2B (see also Burt and Swanepoel^72^). For example, while CCHF occurrences have been reported in central Africa and therefore consensus on presence is relatively high for some countries such as the Democratic Republic of the Congo, the areas of predicted suitability within this region are actually quite sparsely distributed.

Our models showed CCHF risk to be particularly determined by the proportion of grass and shrub land cover within a 5 km×5 km grid cell, contributing 62% to the ensemble of models. Land surface temperature had the second most important effect, contributing 19% to the models, followed by the standard deviation of mean EVI (8%), mean annual precipitation (6%), and mean EVI (5%). Effect plots for each covariate are provided in Supplementary Figure 2. Validation statistics indicated high predictive performance of the BRT ensemble mean map with area under the receiver operating characteristic (AUC) of 0.923 (±0.051 SD).

## Discussion

Temperature, precipitation and moisture indices have been found to be important drivers of CCHF infection in past studies^29, 34^; however, in this model, land cover types were more important in predicting the global ecological niche for CCHF transmission to humans. When considered together, these land cover types are generally reflective of the environments where wild and/or domestic herbivore CCHF hosts exist and enable tick survival and virus circulation. While shrub and grass land cover types are effective for delineating global risk patterns, variations in climate and moisture availability may be more important in predicting heterogeneity in finer-scale prevalence patterns. It is also well understood that those living or working in close proximity to livestock are at the greatest risk for infection with CCHF, yet livestock population layers had minimal influence in predicting human disease occurrence. It is, therefore, possible that the abundance of CCHF livestock reservoirs is more important in driving prevalence patterns within endemic areas rather than as a predictor of overall transmission at a global scale.

We have strived to be exhaustive in the assembly of contemporary data on CCHF occurrence and have applied new modeling approaches to maximise the predictive power of these data. The consideration of a range of biogeographic factors alongside disease occurrence information enabled us to infer risk in areas with uncertainty about CCHF transmission, and the resulting map thus presents sub-national refinements of the distribution of risk without relying on national-level reporting systems. Furthermore, the use of an evidence consensus layer allowed us to limit our predictions to within the current CCHF transmission extents in Africa, Europe and Asia. Due to phylogeographic evidence that CCHFV genotypes tend to vary between Africa and Eurasia more so than within each region,^73^ we did carry out an additional model which distinguished between these two regions. This distinction had a minimal effect and was thus excluded in our final modeling procedures. Such a finding, however, does highlight that while particular CCHFV strains may vary between Africa and Eurasia, the ecological determinants of its zoonotic niche are consistent between the two regions.

**Figure 5.**
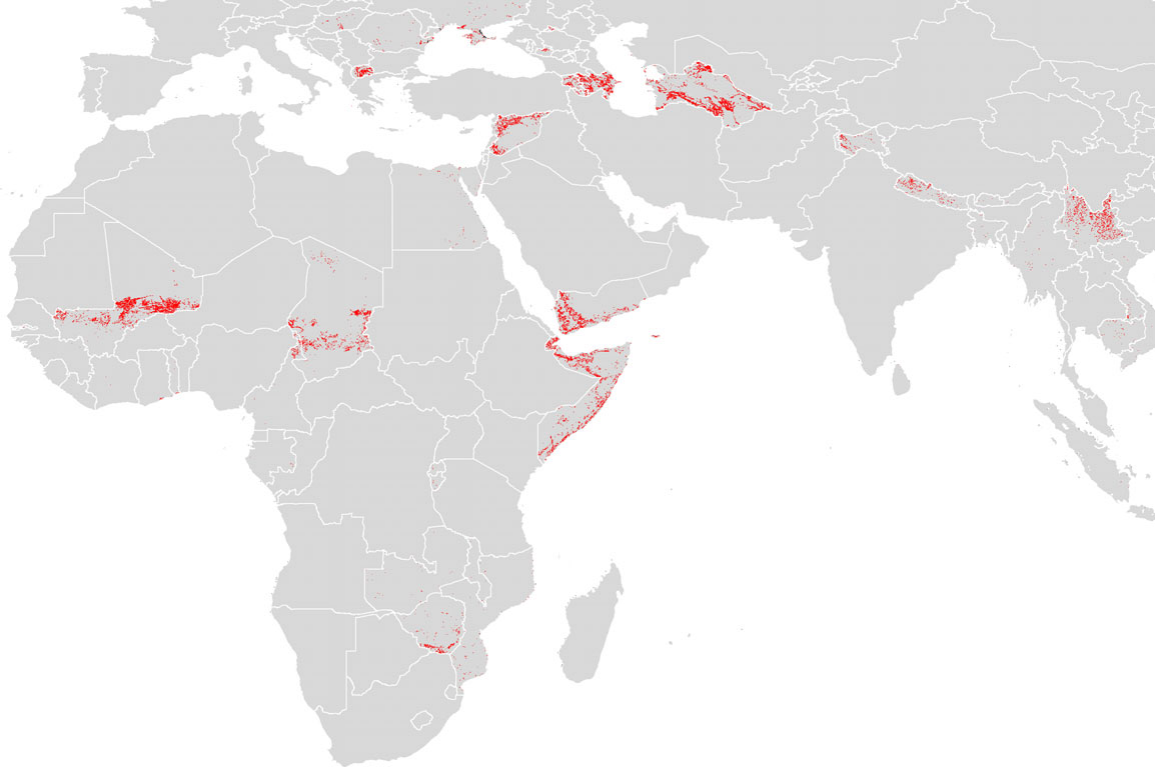
Areas in need of surveillance for Crimean-Congo haemorrhagic fever (CCHF). Red colouring shows areas where our models have predicted high risk for CCHF (≥0.5), but which lie within countries having low evidence consensus (between −25 and +25) on disease presence or absence. The red areas thus signify places most in need of CCHF surveillance.

It is possible to highlight areas where surveillance is most needed. The map in Figure 5 was created by defining ‘high-risk’ pixels as those for which the modelled probability of occurrence (Figure 2C) was greater than or equal to 0.5. We then selected those high-risk pixels which fell inside countries with low evidence consensus scores (between −25 and +25). The result is a visualization of areas where humans are predicted to be at potential risk for CCHF yet where evidence is most lacking and thus where surveillance is a priority. While there are many countries in Africa and Eurasia that have small and sparse areas in need of surveillance, several countries in Figure 5 stand out as having large and more continuous areas in need of surveillance. These include Mali, Chad, Somalia, Djibouti and Zimbabwe in Africa; Syria, Macedonia, Azerbaijian, Armenia, Turkmenistan and Yemen in Eastern Europe and Central Asia; and Kashmir, Nepal and the Yunnan province of China in southern Asia.

For all viral hemorrhagic fevers, public health education about disease vectors or reservoirs and behavioural risk factors for secondary infection is required in endemic areas, and many lessons can be learned from past Marburg, Ebola and Lassa fever outbreaks, for example.^79–81^ Specific to CCHF is the need for agricultural workers and others working with animals to apply acaricidal repellent to exposed skin and clothing, as well as to wear protective clothing and gloves while dealing with the blood or body fluids of livestock. Crimean-Congo hemorrhagic fever does, however, share with these other viral hemorrhagic fevers the risk for infection through contact with other infected humans, and failure to avoid such contact has led to multiple nosocomial CCHF outbreaks in recent years.^82–86^ Such outbreaks indicate that awareness is lacking in many parts of the world about the presence of CCHF, which represents the largest barrier to the rapid diagnosis required for the prevention of nosocomial outbreaks. Healthcare workers in at-risk areas must not only understand the intensive care needs of infected patients, but also understand the precautions required to prevent occupational exposure to CCHF when treating these patients and handling infectious laboratory specimens.^87^ Preventing primary transmission of CCHF to humans also requires strategic allocation of vector and/or reservoir control resources, which are limited in many of the settings we have predicted to be at risk for CCHF, particularly in Africa.

While we have emphasized that better information is still needed in several regions, our map provides a baseline for monitoring change in the global distribution of CCHF going forward. Further cartographic refinements are required in order to help differentiate endemic from epidemic-prone areas, particularly in Africa where there is less certainty about the presence or absence of CCHF overall. The occurrence database used in creating this map can be updated with new information as necessary, and a stronger global evidence base, particularly for the regions highlighted in Figure 5, would improve the accuracy of future iterations of this mapping procedure. The ultimate aim is to provide more useful information in evaluating control and prevention strategies and their impact, and a refined map of the global risk for CCHF is a first step towards reaching this goal.

Although our resulting map is an improvement on those which have previously been produced, the abundance of information about CCHF occurrence still comprises a weaker evidence base than that available, for example, for *Plasmodium falciparum*^74^ and *P. vivax* malaria,^75^ for which a large amount of prevalence information is available. Records of disease occurrence do not easily translate to population-level metrics, and so as databases of CCHF prevalence become more widespread, future approaches should focus on using geostatistical methods to assess risk,^76^ as with many other neglected tropical diseases.^77,78^

### Conclusions

In this study, we have refined the map of the geographic extents of CCHF and the level of risk within these extents using an exhaustive assembly of known records of CCHF occurrence worldwide and an ecological niche modeling framework. We hope that our improved estimate of the spatial distribution of CCHF will serve as a starting point for a wider discussion about the global impact of CCHF. Not only can it encourage public health awareness in areas we have defined as having a high probability of risk, but it can also guide targeted distribution should an effective vaccine or treatment become available.

## Supplementary data

Supplementary data are available at Transactions online (http://trstmh.oxfordjournals.org/).

Supplementary Data
