# Supplementary material for: The global distribution of Crimean-Congo hemorrhagic fever
Source: Trans R Soc Trop Med Hyg. 2015 Jul 4;109(8):503–13. doi: 10.1093/trstmh/trv050 (PMC4501401; doi:10.1093/trstmh/trv050)
Supplement: Supplementary Data [file supp_109_8_503__index.html]

The global distribution of Crimean-Congo hemorrhagic fever — The global distribution of Crimean-Congo hemorrhagic fever — Supplementary Data 

# The global distribution of Crimean-Congo hemorrhagic fever

## Supplementary Data

Supplementary Data

- Supplementary Figure 1 - pdf file
- Supplementary Figure 2 - pdf file
- Supplementary Table 1 - docx file
