# Supplementary material for: The global distribution of Crimean-Congo hemorrhagic fever
Source: Trans R Soc Trop Med Hyg. 2015 Jul 4;109(8):503–13. doi: 10.1093/trstmh/trv050 (PMC4501401; doi:10.1093/trstmh/trv050)
Supplement: Supplementary Data [file supp_trv050_trv050supp_fig1.pdf]

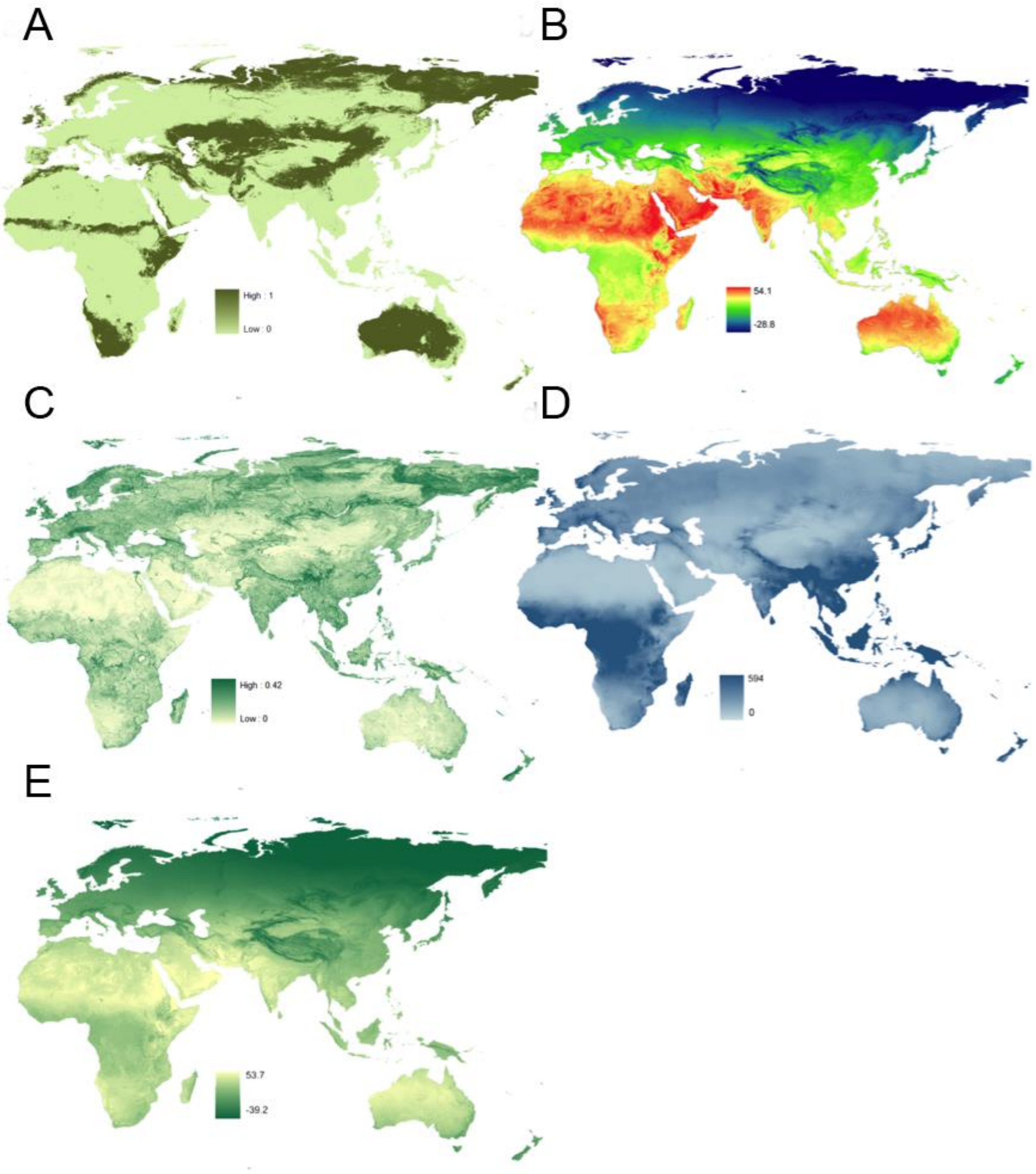

Supplementary Figure 1. Plots of all covariates entered into the boosted regression tree (BRT).

A: % grass or shrub land cover; B: mean annual land surface temperature; C: standard deviation of mean annual EVI; D: mean annual precipitation (mm); E: mean annual EVI
