# Supplementary material for: The global distribution of Crimean-Congo hemorrhagic fever
Source: Trans R Soc Trop Med Hyg. 2015 Jul 4;109(8):503–13. doi: 10.1093/trstmh/trv050 (PMC4501401; doi:10.1093/trstmh/trv050)
Supplement: Supplementary Data [file supp_trv050_trv050supp_table1.docx]

**Supplementary Table 1.** Evidence consensus scoring for Crimean-Congo haemorrhagic fever

|  | GIDEON status | WHO status | HOS | Peer-reviewed evidence | LS | Case score | HES | Supplementary information | SIS | Final score |
| --- | --- | --- | --- | --- | --- | --- | --- | --- | --- | --- |
| Countries | | | | | | | | | | |
| Afghanistan | Endemic | 5–49 cases | 2.5 | PCR, 2012[^1^](#_ENREF_1) | 6 | 6 | Medium | NA | NA | 96.67 |
| Albania | Endemic | 5–49 cases | 2.5 | PCR, 2001[^2^](#_ENREF_2) | 5 | 4 | Medium | NA | NA | 76.67 |
| Algeria | Not endemic | *Hyalomma* ticks | -2 | NA | NA | -2 | Medium | NA | NA | -44.44 |
| American Samoa | Not endemic | Absent | -3 | NA | NA | -6 | High | NA | NA | -100.00 |
| Andorra | Not endemic | *Hyalomma* ticks | -2 | NA | NA | -6 | High | NA | NA | -88.89 |
| Angola | Not endemic | *Hyalomma* ticks | -2 | NA | NA | -2 | Medium | NA | NA | -44.44 |
| Anguilla | Not endemic | Absent | -3 | NA | NA | -6 | High | NA | NA | -100.00 |
| Antarctica | Not endemic | Absent | -3 | NA | NA | -2 | Medium | NA | NA | -55.56 |
| Antigua and Barbuda | Not endemic | Absent | -3 | NA | NA | -6 | High | NA | NA | -100.00 |
| Argentina | Not endemic | Absent | -3 | NA | NA | -6 | High | NA | NA | -100.00 |
| Armenia | Endemic | Viro/sero evidence | 2 | NA | NA | -2 | Medium | NA | NA | 0.00 |
| Aruba | Not endemic | Absent | -3 | NA | NA | -6 | High | NA | NA | -100.00 |
| Australia | Not endemic | Absent | -3 | NA | NA | -6 | High | NA | NA | -100.00 |
| Austria | Not endemic | *Hyalomma* ticks | -2 | NA | NA | -6 | High | NA | NA | -88.89 |
| Azerbaijan | Endemic | Viro/sero evidence | 2 | NA | NA | -2 | Medium | NA | NA | 0.00 |
| Azores Islands | Not endemic | Absent | -3 | NA | NA | -6 | High | NA | NA | -100.00 |
| Bahamas | Not endemic | Absent | -3 | NA | NA | -6 | High | NA | NA | -100.00 |
| Bahrain | Not endemic | Absent | -3 | NA | NA | -6 | High | NA | NA | -100.00 |
| Baker Island | Unspecified | Absent | -2 | NA | NA | -6 | High | NA | NA | -88.89 |
| Bangladesh | Not endemic | Viro/sero evidence | -1.5 | NA | NA | 2 | Low | NA | NA | 5.56 |
| Barbados | Not endemic | Absent | -3 | NA | NA | -6 | High | NA | NA | -100.00 |
| Belarus | Endemic | Absent | 0 | NA | NA | -2 | Medium | NA | NA | -22.22 |
| Belgium | Not endemic | Absent | -3 | NA | NA | -6 | High | NA | NA | -100.00 |
| Belize | Not endemic | Absent | -3 | NA | NA | -2 | Medium | NA | NA | -55.56 |
| Benin | Endemic | Viro/sero evidence | 2 | NA | NA | 2 | Low | NA | NA | 44.44 |
| Bermuda | Not endemic | Absent | -3 | NA | NA | -6 | High | NA | NA | -100.00 |
| Bhutan | Not endemic | *Hyalomma* ticks | -2 | NA | NA | 2 | Low | NA | NA | 0.00 |
| Bird Island | Unspecified | Absent | -2 | NA | NA | -6 | High | NA | NA | -88.89 |
| Bolivia | Not endemic | Absent | -3 | NA | NA | -2 | Medium | NA | NA | -55.56 |
| Bosnia and Herzegovina | Not endemic | Viro/sero evidence | -1.5 | NA | NA | -2 | Medium | NA | NA | -38.89 |
| Botswana | Not endemic | Viro/sero evidence | -1.5 | NA | NA | -2 | Medium | NA | NA | -38.89 |
| Bouvet Island | Unspecified | Absent | -2 | NA | NA | -6 | High | NA | NA | -88.89 |
| Brazil | Not endemic | Absent | -3 | NA | NA | -6 | High | NA | NA | -100.00 |
| British Indian Ocean Territory | Unspecified | Absent | -2 | NA | NA | -6 | High | NA | NA | -88.89 |
| British Virgin Islands | Not endemic | Absent | -3 | NA | NA | -6 | High | NA | NA | -100.00 |
| Brunei Darussalam | Not endemic | Absent | -3 | NA | NA | -6 | High | NA | NA | -100.00 |
| Bulgaria | Endemic | 5–49 cases | 2.5 | 2008, PCR[^3^](#_ENREF_3) | 6 | 4 | Medium | NA | NA | 83.33 |
| Burkina Faso | Endemic | Viro/sero evidence | 2 | NA | NA | 2 | Low | NA | NA | 44.44 |
| Burundi | Not endemic | *Hyalomma* ticks | -2 | NA | NA | 2 | Low | NA | NA | 0.00 |
| Cambodia | Not endemic | *Hyalomma* ticks | -2 | NA | NA | 2 | Low | NA | NA | 0.00 |
| Cameroon | Not endemic | Viro/sero evidence | -1.5 | NA | NA | 2 | Low | Human seroprevalence 1985–1987[^4^](#_ENREF_4) | 15 | 20.56 |
| Canada | Not endemic | Absent | -3 | NA | NA | -6 | High | NA | NA | -100.00 |
| Cape Verde | Not endemic | Absent | -3 | NA | NA | 2 | Low | NA | NA | -11.11 |
| Cayman Islands | Not endemic | Absent | -3 | NA | NA | -6 | High | NA | NA | -100.00 |
| Central African Republic | Endemic | Viro/sero evidence | 2 | NA | NA | 2 | Low | NA | NA | 44.44 |
| Chad | Not endemic | *Hyalomma* ticks | -2 | NA | NA | 2 | Low | Human seroprevalence 1985–1987[^4^](#_ENREF_4) | 15 | 15.00 |
| Chile | Not endemic | Absent | -3 | NA | NA | -6 | High | NA | NA | -100.00 |
| China | Endemic | 5–49 cases | 2.5 | NA | 5 | 4 | Medium | NA | NA | 76.67 |
| China/India | Unspecified | Absent | -2 | NA | NA | -2 | Medium | NA | NA | -44.44 |
| Christmas Island | Not endemic | Absent | -3 | NA | NA | -6 | High | NA | NA | -100.00 |
| Colombia | Not endemic | Absent | -3 | NA | NA | -2 | Medium | NA | NA | -55.56 |
| Comoros | Not endemic | Absent | -3 | NA | NA | 2 | Low | NA | NA | -11.11 |
| Congo | Endemic | Viro/sero evidence | 2 | NA | NA | 2 | Low | NA | NA | 44.44 |
| Cocos (Keeling) Islands | Unspecified | Absent | -2 | NA | NA | -6 | High | NA | NA | -88.89 |
| Cook Islands | Not endemic | Absent | -3 | NA | NA | -6 | High | NA | NA | -100.00 |
| Costa Rica | Not endemic | Absent | -3 | NA | NA | -6 | High | NA | NA | -100.00 |
| Côte d'Ivoire | Not endemic | Viro/sero evidence | -1.5 | NA | NA | 2 | Low | NA | NA | 5.56 |
| Croatia | Not endemic | Viro/sero evidence | -1.5 | NA | NA | -6 | High | NA | NA | -83.33 |
| Cuba | Not endemic | Absent | -3 | NA | NA | -6 | High | NA | NA | -100.00 |
| Cyprus | Not endemic | *Hyalomma* ticks | -2 | NA | NA | -6 | High | NA | NA | -88.89 |
| Czech Republic | Not endemic | Absent | -3 | NA | NA | -6 | High | NA | NA | -100.00 |
| Democratic People's Rep of Korea | Not endemic | *Hyalomma* ticks | -2 | 2001, Reported |  | -2 | Medium | NA | NA | -44.44 |
| Democratic Republic of the Congo | Endemic | Viro/sero evidence | 2 | 2001, Reported | 6 | 2 | Low | NA | NA | 66.67 |
| Djibouti | Not endemic | *Hyalomma* ticks | -2 | NA | NA | 2 | Low | Sheep and goats, sero-prevalence[^5^](#_ENREF_5) | 10 | 10.00 |
| Dominica | Not endemic | Absent | -3 | NA | NA | -2 | Medium | NA | NA | -55.56 |
| Dominican Republic | Not endemic | Absent | -3 | NA | NA | -2 | Medium | NA | NA | -55.56 |
| Ecuador | Not endemic | Absent | -3 | NA | NA | -2 | Medium | NA | NA | -55.56 |
| Egypt | Endemic | Viro/sero evidence | 2 | NA | NA | -2 | Medium | NA | NA | 0.00 |
| El Salvador | Not endemic | Absent | -3 | NA | NA | -2 | Medium | NA | NA | -55.56 |
| Equatorial Guinea | Not endemic | *Hyalomma* ticks | -2 | NA | NA | -6 | High | Human seroprevalence 1985–1987[^4^](#_ENREF_4) | 15 | -73.89 |
| Eritrea | Endemic | *Hyalomma* ticks | 1.5 | NA | NA | 2 | Low | NA | NA | 38.89 |
| Estonia | Endemic | Absent | 0 | NA | NA | -6 | High | NA | NA | -66.67 |
| Ethiopia | Endemic | Viro/sero evidence | 2 | NA | NA | 2 | Low | Tick, 1975[^6^](#_ENREF_6) | 5 | 49.44 |
| Europa Island | Unspecified | Absent | -2 | NA | NA | -2 | Medium | NA | NA | -44.44 |
| Falkland Islands (Malvinas) | Not endemic | Absent | -3 | NA | NA | -6 | High | NA | NA | -100.00 |
| Faroe Islands | Unspecified | Absent | -2 | NA | NA | -6 | High | NA | NA | -88.89 |
| Fiji | Not endemic | Absent | -3 | NA | NA | -2 | Medium | NA | NA | -55.56 |
| Finland | Not endemic | Absent | -3 | NA | NA | -6 | High | NA | NA | -100.00 |
| France | Not endemic | *Hyalomma* ticks | -2 | NA | NA | -6 | High | NA | NA | -88.89 |
| French Guiana | Not endemic | Absent | -3 | NA | NA | -6 | High | NA | NA | -100.00 |
| French Polynesia | Not endemic | Absent | -3 | NA | NA | -6 | High | NA | NA | -100.00 |
| French Southern and Antarctic Territories | Not endemic | Absent | -3 | NA | NA | -6 | High | NA | NA | -100.00 |
| Gabon | Not endemic | Viro/sero evidence | -1.5 | NA | NA | -2 | Medium | Human seroprevalence 1985–1987[^4^](#_ENREF_4) | 15 | -23.89 |
| Gambia | Not endemic | Viro/sero evidence | -1.5 | NA | NA | 2 | Low | NA | NA | 5.56 |
| Georgia | Endemic | Viro/sero evidence | 2 | 2009, PCR[^7^](#_ENREF_7) | 6 | -2 | Medium | NA | NA | 40.00 |
| Germany | Not endemic | *Hyalomma* ticks | -2 | NA | NA | -6 | High | NA | NA | -88.89 |
| Ghana | Not endemic | *Hyalomma* ticks | -2 | NA | NA | 2 | Low | NA | NA | 0.00 |
| Gibraltar | Not endemic | *Hyalomma* ticks | -2 | NA | NA | -6 | High | NA | NA | -88.89 |
| Glorioso Island | Unspecified | Absent | -2 | NA | NA | -6 | High | NA | NA | -88.89 |
| Greece | Endemic | Viro/sero evidence | 2 | 2008, PCR[^8^](#_ENREF_8) | 6 | -6 | High | Human seroprevalence 1978-2013[^9^](#_ENREF_9)^,^[^10^](#_ENREF_10)^,^[^11^](#_ENREF_11) | 15 | 28.33 |
| Greenland | Not endemic | Absent | -3 | NA | NA | -6 | High | NA | NA | -100.00 |
| Grenada | Not endemic | Absent | -3 | NA | NA | -2 | Medium | NA | NA | -55.56 |
| Guadeloupe | Not endemic | Absent | -3 | NA | NA | -6 | High | NA | NA | -100.00 |
| Guam | Not endemic | Absent | -3 | NA | NA | -6 | High | NA | NA | -100.00 |
| Guatemala | Not endemic | Absent | -3 | NA | NA | -2 | Medium | NA | NA | -55.56 |
| Guernsey | Not endemic | Absent | -3 | NA | NA | -6 | High | NA | NA | -100.00 |
| Guinea | Not endemic | Viro/sero evidence | -1.5 | NA | NA | 2 | Low | Ticks, 1978–1991[^12^](#_ENREF_12) | 5 | 10.56 |
| Guinea-Bissau | Not endemic | Viro/sero evidence | -1.5 | NA | NA | 2 | Low | NA | NA | 5.56 |
| Guyana | Not endemic | Absent | -3 | NA | NA | -2 | Medium | NA | NA | -55.56 |
| Haiti | Not endemic | Absent | -3 | NA | NA | 2 | Low | NA | NA | -11.11 |
| Heard Island and McDonald Islands | Unspecified | Absent | -2 | NA | NA | -6 | High | NA | NA | -88.89 |
| Holy See | Not endemic | *Hyalomma* ticks | -2 | NA | NA | -6 | High | NA | NA | -88.89 |
| Honduras | Not endemic | Absent | -3 | NA | NA | -2 | Medium | NA | NA | -55.56 |
| Hong Kong | Not endemic | *Hyalomma* ticks | -2 | NA | NA | -6 | High | NA | NA | -88.89 |
| Howland Island | Unspecified | Absent | -2 | NA | NA | -6 | High | NA | NA | -88.89 |
| Hungary | Endemic | Viro/sero evidence | 2 | 2004, Reported[^13^](#_ENREF_13) | 3 | -6 | High | Human seroprevalence, 1976[^14^](#_ENREF_14) | 15 | 8.33 |
| Iceland | Not endemic | Absent | -3 | NA | NA | -6 | High | NA | NA | -100.00 |
| India | Endemic | Viro/sero evidence | 2 | 2011, PCR[^15^](#_ENREF_15) | 6 | 6 | Low | NA | NA | 93.33 |
| Indonesia | Not endemic | Absent | -3 | NA | NA | 2 | Low | NA | NA | -11.11 |
| Iran (Islamic Republic of) | Endemic | 50+ cases | 3 | 2012, PCR[^16^](#_ENREF_16) | 6 | 6 | Medium | NA | NA | 100.00 |
| Iraq | Endemic | Viro/sero evidence | 2 | 2010, Serological[^17^](#_ENREF_17) | 5 | 6 | Medium | NA | NA | 86.67 |
| Ireland | Not endemic | Absent | -3 | NA | NA | -6 | High | NA | NA | -100.00 |
| Israel | Not endemic | *Hyalomma* ticks | -2 | NA | NA | -6 | High | NA | NA | -88.89 |
| Italy | Not endemic | *Hyalomma* ticks | -2 | NA | NA | -6 | High | NA | NA | -88.89 |
| Jamaica | Not endemic | Absent | -3 | NA | NA | -2 | Medium | NA | NA | -55.56 |
| Jammu Kashmir | Unspecified | Absent | -2 | - | 5 | 2 | Low | NA | NA | 33.33 |
| Japan | Not endemic | Absent | -3 | NA | NA | -6 | High | NA | NA | -100.00 |
| Jarvis Island | Unspecified | Absent | -2 | NA | NA | -6 | High | NA | NA | -88.89 |
| Jersey | Not endemic | Absent | -3 | NA | NA | -6 | High | NA | NA | -100.00 |
| Jordan | Not endemic | *Hyalomma* ticks | -2 | NA | NA | -2 | Medium | NA | NA | -44.44 |
| Juan de Nova Island | Unspecified | Absent | -2 | NA | NA | -6 | High | NA | NA | -88.89 |
| Kazakhstan | Endemic | 5–49 cases | 2.5 | 2003, PCR[^18^](#_ENREF_18) | 5 | 6 | Medium | NA | NA | 90.00 |
| Kenya | Endemic | Viro/sero evidence | 2 | 2000, PCR[^19^](#_ENREF_19) | 5 | 2 | Low | NA | NA | 60.00 |
| Kiribati | Not endemic | Absent | -3 | NA | NA | -2 | Medium | NA | NA | -55.56 |
| Korea, Republic of | Not endemic | *Hyalomma* ticks | -2 | NA | NA | -6 | High | NA | NA | -88.89 |
| Kuwait | Endemic | Absent | 0 | NA | NA | -6 | High | NA | NA | -66.67 |
| Kyrgyzstan | Endemic | 5–49 cases | 2.5 | 1979, reported[^20^](#_ENREF_20) | 2 | 2 | Low | NA | NA | 43.33 |
| Lao People's Democratic Republic | Not endemic | *Hyalomma* ticks | -2 | NA | NA | 2 | Low | NA | NA | 0.00 |
| Latvia | Endemic | Absent | 0 | NA | NA | -6 | High | NA | NA | -66.67 |
| Lebanon | Not endemic | *Hyalomma* ticks | -2 | NA | NA | -6 | High | NA | NA | -88.89 |
| Lesotho | Not endemic | Viro/sero evidence | -1.5 | NA | NA | -2 | Medium | NA | NA | -38.89 |
| Liancourt Rock | Unspecified | Absent | -2 | NA | NA | -6 | High | NA | NA | -88.89 |
| Liberia | Not endemic | *Hyalomma* ticks | -2 | NA | NA | 2 | Low | NA | NA | 0.00 |
| Libyan Arab Jamahiriya | Not endemic | *Hyalomma* ticks | -2 | NA | NA | -2 | Medium | NA | NA | -44.44 |
| Liechtenstein | Not endemic | *Hyalomma* ticks | -2 | NA | NA | -2 | Medium | NA | NA | -44.44 |
| Lithuania | Endemic | Absent | 0 | NA | NA | -6 | High | NA | NA | -66.67 |
| Luxembourg | Not endemic | Absent | -3 | NA | NA | -6 | High | NA | NA | -100.00 |
| Macau | Not endemic | *Hyalomma* ticks | -2 | NA | NA | -2 | Medium | NA | NA | -44.44 |
| Madagascar | Endemic | Viro/sero evidence | 2 | NA | NA | 2 | Low | NA | NA | 44.44 |
| Madeira Islands | Unspecified | Absent | -2 | NA | NA | -6 | High | NA | NA | -88.89 |
| Malawi | Not endemic | *Hyalomma* ticks | -2 | NA | NA | 2 | Low | NA | NA | 0.00 |
| Malaysia | Not endemic | Absent | -3 | NA | NA | -2 | Medium | NA | NA | -55.56 |
| Maldives | Not endemic | Absent | -3 | NA | NA | -2 | Medium | NA | NA | -55.56 |
| Mali | Not endemic | *Hyalomma* ticks | -2 | NA | NA | 2 | Low | NA | NA | 0.00 |
| Malta | Not endemic | Absent | -3 | NA | NA | -6 | High | NA | NA | -100.00 |
| Marshall Islands | Not endemic | Absent | -3 | NA | NA | -6 | High | NA | NA | -100.00 |
| Martinique | Not endemic | Absent | -3 | NA | NA | -6 | High | NA | NA | -100.00 |
| Mauritania | Endemic | Viro/sero evidence | 2 | 2003, PCR[^21^](#_ENREF_21) | 5 | 4 | Low | NA | NA | 73.33 |
| Mauritius | Not endemic | Absent | -3 | NA | NA | -2 | Medium | NA | NA | -55.56 |
| Mayotte | Unspecified | Absent | -2 | NA | NA | -6 | High | NA | NA | -88.89 |
| Mexico | Not endemic | Absent | -3 | NA | NA | -6 | High | NA | NA | -100.00 |
| Micronesia (Federated States of) | Not endemic | Absent | -3 | NA | NA | -2 | Medium | NA | NA | -55.56 |
| Midway Island | Unspecified | Absent | -2 | NA | NA | -6 | High | NA | NA | -88.89 |
| Monaco | Not endemic | *Hyalomma* ticks | -2 | NA | NA | -6 | High | NA | NA | -88.89 |
| Mongolia | Not endemic | *Hyalomma* ticks | -2 | NA | NA | -2 | Medium | NA | NA | -44.44 |
| Montenegro | Endemic | 5–49 cases | 2.5 | NA | NA | -6 | High | NA | NA | -38.89 |
| Montserrat | Not endemic | Absent | -3 | NA | NA | -6 | High | NA | NA | -100.00 |
| Morocco | Not endemic | *Hyalomma* ticks | -2 | NA | NA | -2 | Medium | Ticks 2011[^22^](#_ENREF_22) | 5 | -39.44 |
| Mozambique | Not endemic | *Hyalomma* ticks | -2 | NA | NA | 2 | Low | NA | NA | 0.00 |
| Myanmar | Not endemic | *Hyalomma* ticks | -2 | NA | NA | 2 | Low | NA | NA | 0.00 |
| Namibia | Endemic | Viro/sero evidence | 2 | 2001, PCR[^23^](#_ENREF_23) | 5 | -2 | Medium | NA | NA | 33.33 |
| Nauru | Not endemic | Absent | -3 | NA | NA | -6 | High | NA | NA | -100.00 |
| Navassa Island | Unspecified | Absent | -2 | NA | NA | -6 | High | NA | NA | -88.89 |
| Nepal | Not endemic | *Hyalomma* ticks | -2 | NA | NA | 2 | Low | NA | NA | 0.00 |
| Netherlands | Not endemic | Absent | -3 | NA | NA | -6 | High | NA | NA | -100.00 |
| Netherlands Antilles | Not endemic | Absent | -3 | NA | NA | -6 | High | NA | NA | -100.00 |
| New Caledonia | Not endemic | Absent | -3 | NA | NA | -6 | High | NA | NA | -100.00 |
| New Zealand | Not endemic | Absent | -3 | NA | NA | -6 | High | NA | NA | -100.00 |
| Nicaragua | Not endemic | Absent | -3 | NA | NA | -2 | Medium | NA | NA | -55.56 |
| Niger | Endemic | *Hyalomma* ticks | 1.5 | NA | NA | 2 | Low | Animal: cattle, sheep, goats, camels, small ruminants, 1985–1988[^24^](#_ENREF_24) | 10 | 48.89 |
| Nigeria | Endemic | Viro/sero evidence | 2 | NA | NA | 2 | Low | Ticks, 1966[^25^](#_ENREF_25) | 5 | 49.44 |
| Niue | Not endemic | Absent | -3 | NA | NA | -6 | High | NA | NA | -100.00 |
| Norway | Not endemic | Absent | -3 | NA | NA | -6 | High | NA | NA | -100.00 |
| Norfolk Island | Not endemic | Absent | -3 | NA | NA | -6 | High | NA | NA | -100.00 |
| Northern Mariana Islands | Not endemic | Absent | -3 | NA | NA | -6 | High | NA | NA | -100.00 |
| Oman | Endemic | Viro/sero evidence | 2 | 2011, PCR[^26^](#_ENREF_26) | 6 | -6 | High | Human seroprevalence, 1995–1996[^27^](#_ENREF_27) | 15 | 28.33 |
| Pakistan | Endemic | 5–49 cases | 2.5 | 2012, PCR[^28^](#_ENREF_28) | 6 | 6 | Low | NA | NA | 96.67 |
| Palau | Not endemic | Absent | -3 | NA | NA | -6 | High | NA | NA | -100.00 |
| Palmyra Atoll | Unspecified | Absent | -2 | NA | NA | -6 | High | NA | NA | -88.89 |
| Panama | Not endemic | Absent | -3 | NA | NA | -6 | High | NA | NA | -100.00 |
| Papua New Guinea | Not endemic | Absent | -3 | NA | NA | 2 | Low | NA | NA | -11.11 |
| Paraguay | Not endemic | Absent | -3 | NA | NA | -2 | Medium | NA | NA | -55.56 |
| Peru | Not endemic | Absent | -3 | NA | NA | -2 | Medium | NA | NA | -55.56 |
| Philippines | Not endemic | Absent | -3 | NA | NA | 2 | Low | NA | NA | -11.11 |
| Pitcairn | Not endemic | Absent | -3 | NA | NA | -6 | High | NA | NA | -100.00 |
| Poland | Not endemic | Absent | -3 | NA | NA | -6 | High | NA | NA | -100.00 |
| Portugal | Not endemic | *Hyalomma* ticks | -2 | NA | NA | -6 | High | Human seroprevalence, 1985[^29^](#_ENREF_29) | 15 | -73.89 |
| Puerto Rico | Not endemic | Absent | -3 | NA | NA | -6 | High | NA | NA | -100.00 |
| Qatar | Not endemic | Absent | -3 | NA | NA | -6 | High | NA | NA | -100.00 |
| Republic of Moldova | Endemic | Viro/sero evidence | 2 | NA | NA | -2 | Medium | NA | NA | 0.00 |
| Romania | Endemic | Viro/sero evidence | 2 | NA | NA | -2 | Medium | Sheep, 2008[^30^](#_ENREF_30) | 10 | 10.00 |
| Rwanda | Not endemic | *Hyalomma* ticks | -2 | NA | NA | 2 | Low | NA | NA | 0.00 |
| Saint Helena | Not endemic | Absent | -3 | NA | NA | -6 | High | NA | NA | -100.00 |
| Saint Kitts and Nevis | Not endemic | Absent | -3 | NA | NA | -6 | High | NA | NA | -100.00 |
| Saint Lucia | Not endemic | Absent | -3 | NA | NA | -6 | High | NA | NA | -100.00 |
| Saint Pierre et Miquelon | Unspecified | Absent | -2 | NA | NA | -6 | High | NA | NA | -88.89 |
| Saint Vincent and the Grenadines | Not endemic | Absent | -3 | NA | NA | -2 | Medium | NA | NA | -55.56 |
| Samoa | Not endemic | Absent | -3 | NA | NA | -2 | Medium | NA | NA | -55.56 |
| San Marino | Not endemic | *Hyalomma* ticks | -2 | NA | NA | -6 | High | NA | NA | -88.89 |
| Sao Tome and Principe | Not endemic | Absent | -3 | NA | NA | 2 | Low | NA | NA | -11.11 |
| Saudi Arabia | Endemic | Viro/sero evidence | 2 | 1992, Serological[^31^](#_ENREF_31) | 3 | 2 | High | NA | NA | 46.67 |
| Scarborough Reef | Unspecified | Absent | -2 | 1992, Serological[^31^](#_ENREF_31) |  | -2 | Medium | NA | NA | -44.44 |
| Senegal | Endemic | Viro/sero evidence | 2 | 2004, PCR[^32^](#_ENREF_32) | 5 | 2 | Low | NA | NA | 60.00 |
| Senkaku Islands | Unspecified | Absent | -2 | NA | NA | -6 | High | NA | NA | -88.89 |
| Serbia | Endemic | 5–49 cases | 2.5 | 2005, PCR; 2009, Serological^[33](#_ENREF_33)^ | 5 | 6 | High | NA | NA | 90.00 |
| Seychelles | Not endemic | Absent | -3 | NA | NA | -2 | Medium | NA | NA | -55.56 |
| Sierra Leone | Not endemic | *Hyalomma* ticks | -2 | NA | NA | 2 | Low | NA | NA | 0.00 |
| Singapore | Not endemic | Absent | -3 | NA | NA | -6 | High | NA | NA | -100.00 |
| Slovakia | Not endemic | *Hyalomma* ticks | -2 | NA | NA | -6 | High | NA | NA | -88.89 |
| Slovenia | Not endemic | *Hyalomma* ticks | -2 | NA | NA | -6 | High | NA | NA | -88.89 |
| Solomon Islands | Not endemic | Absent | -3 | NA | NA | 2 | Low | NA | NA | -11.11 |
| Somalia | Not endemic | *Hyalomma* ticks | -2 | NA | NA | 2 | Low | NA | NA | 0.00 |
| South Africa | Endemic | 5–49 cases | 2.5 | 2006, PCR[^34^](#_ENREF_34) | 6 | 4 | High | NA | NA | 83.33 |
| South Georgia and the South Sandwich Islands | Unspecified | Absent | -2 | 2006, PCR[^34^](#_ENREF_34) |  | -6 | High | NA | NA | -88.89 |
| South Sudan | Endemic | Viro/sero evidence | 2 | NA | NA | 2 | Low | NA | NA | 44.44 |
| Spain | Not endemic | *Hyalomma* ticks | -2 | NA | NA | -6 | High | NA | NA | -88.89 |
| Sri Lanka | Not endemic | Absent | -3 | NA | NA | 2 | Low | NA | NA | -11.11 |
| Sudan | Endemic | Viro/sero evidence | 2 | 2011, PCR[^35^](#_ENREF_35) | 6 | 6 |  | NA | NA | 93.33 |
| Suriname | Not endemic | Absent | -3 | NA | NA | -2 | Medium | NA | NA | -55.56 |
| Svalbard and Jan Mayen Islands | Unspecified | Absent | -2 | NA | NA | -6 | High | NA | NA | -88.89 |
| Swaziland | Not endemic | Viro/sero evidence | -1.5 | NA | NA | -2 | Medium | NA | NA | -38.89 |
| Sweden | Not endemic | Absent | -3 | NA | NA | -6 | High | NA | NA | -100.00 |
| Switzerland | Not endemic | *Hyalomma* ticks | -2 | NA | NA | -6 | High | NA | NA | -88.89 |
| Syrian Arab Republic | Not endemic | Viro/sero evidence | -1.5 | NA | NA | 2 | Low | NA | NA | 5.56 |
| Tajikistan | Endemic | 5–49 cases | 2.5 | 2010, PCR[^1^](#_ENREF_1) | 6 | 6 | Low | NA | NA | 96.67 |
| Thailand | Not endemic | *Hyalomma* ticks | -2 | NA | NA | -2 | Medium | NA | NA | -44.44 |
| The former Yugoslav Republic of Macedonia | Endemic | Viro/sero evidence | 2 | NA | NA | -6 | Medium | NA | NA | -13.33 |
| Timor-Leste | Not endemic | Absent | -3 | NA | NA | 2 | Low | NA | NA | -11.11 |
| Togo | Not endemic | *Hyalomma* ticks | -2 | NA | NA | 2 | Low | NA | NA | 0.00 |
| Tokelau | Not endemic | Absent | -3 | NA | NA | -6 | High | NA | NA | -100.00 |
| Tonga | Not endemic | Absent | -3 | NA | NA | -2 | Medium | NA | NA | -55.56 |
| Trinidad and Tobago | Not endemic | Absent | -3 | NA | NA | -6 | High | NA | NA | -100.00 |
| Tromelin Island | Unspecified | Absent | -2 | NA | NA | -6 | High | NA | NA | -88.89 |
| Tunisia | Not endemic | *Hyalomma* ticks | -2 | NA | NA | -2 | Medium | NA | NA | -44.44 |
| Turkey | Endemic | 50+ cases | 3 | 2007, PCR[^36^](#_ENREF_36) | 6 | 6 | High | NA | NA | 100.00 |
| Turkmenistan | Endemic | Viro/sero evidence | 2 | NA | NA | -2 | Medium | Ticks, 1984[^37^](#_ENREF_37) & 1968–1976[^38^](#_ENREF_38)  Human seroprevalence, 1968–1976[^38^](#_ENREF_38) | 15 | 15.00 |
| Turks and Caicos islands | Not endemic | Absent | -3 | NA | NA | -6 | High | NA | NA | -100.00 |
| Tuvalu | Not endemic | Absent | -3 | NA | NA | -2 | Medium | NA | NA | -55.56 |
| Uganda | Endemic | Viro/sero evidence | 2 | NA | NA | 2 | Low | Human seroprevalence, 1984 [^39^](#_ENREF_39) | 15 | 59.44 |
| Ukraine | Endemic | Viro/sero evidence | 2 | NA | NA | -2 | Medium | Human seroprevalence, Tick, animal: rodents, cattle[^40^](#_ENREF_40) | 15 | 15.00 |
| United Arab Emirates | Endemic | 5–49 cases | 2.5 | 1994, PCR[^41^](#_ENREF_41)^,^[^42^](#_ENREF_42) | 4 | 2 | High | NA | NA | 56.67 |
| United Kingdom of Great Britain and Northern Ireland | Endemic | Absent | 0 | NA | NA | -6 | High | NA | NA | -66.67 |
| United Republic of Tanzania | Endemic | Viro/sero evidence | 2 | NA | NA | 2 | Low | NA | NA | 44.44 |
| United States of America | Not endemic | Absent | -3 | NA | NA | -6 | High | NA | NA | -100.00 |
| United States Virgin Islands | Not endemic | Absent | -3 | NA | NA | -6 | High | NA | NA | -100.00 |
| Uruguay | Not endemic | Absent | -3 | NA | NA | -6 | High | NA | NA | -100.00 |
| Uzbekistan | Endemic | 50+ cases | 3 | 1967, PCR[^43^](#_ENREF_43) | 4 | 2 | Low | NA | NA | 60.00 |
| Vanuatu | Not endemic | Absent | -3 | NA | NA | -2 | Medium | NA | NA | -55.56 |
| Venezuela | Not endemic | Absent | -3 | NA | NA | -6 | High | NA | NA | -100.00 |
| Viet Nam | Not endemic | *Hyalomma* ticks | -2 | NA | NA | 2 | Low | NA | NA | 0.00 |
| Wallis and Futuna | Not endemic | Absent | -3 | NA | NA | -6 | High | NA | NA | -100.00 |
| West Bank | Unspecified | Absent | -2 | NA | NA | -2 | Medium | NA | NA | -44.44 |
| Western Sahara | Not endemic | Absent | -3 | NA | NA | -2 | Medium | NA | NA | -55.56 |
| Yemen | Not endemic | *Hyalomma* ticks | -2 | NA | NA | 2 | Low | NA | NA | 0.00 |
| Zambia | Not endemic | *Hyalomma* ticks | -2 | NA | NA | 2 | Low | NA | NA | 0.00 |
| Zimbabwe | Endemic | Viro/sero evidence | 2 | NA | NA | -2 | Medium | Human seroprevalence^[44](#_ENREF_44)^  Animal: cattle[^45^](#_ENREF_45)  Animal: rhino, dog, giraffe, zebra, oryx, hare, rodent, buffalo, kudo, zebra[^46^](#_ENREF_46) | 15 | 15.00 |
| Provinces and other territories | |  |  |  |  |  |  |  |  |  |
| Adygeya Rep. | Not endemic | 50+ cases | -0.5 | NA | NA | -6 | High | NA | NA | -72.22 |
| Aginskiy Buryatskiy A. Okrug | Not endemic | Absent | -3 | NA | NA | -6 | High | NA | NA | -100.00 |
| Aksai Chin | Unspecified | *Hyalomma* ticks | 0.5 | NA | NA | -2 | Medium | NA | NA | -16.67 |
| Altay Rep. | Not endemic | Absent | -3 | NA | NA | -6 | High | NA | NA | -100.00 |
| Altayskiy Kray | Not endemic | Absent | -3 | NA | NA | -6 | High | NA | NA | -100.00 |
| Amurskaya Oblast | Not endemic | Absent | -3 | NA | NA | -6 | High | NA | NA | -100.00 |
| Anhui Sheng | Not endemic | *Hyalomma* ticks | -2 | NA | NA | -2 | Medium | Human seroprevalence, 2010[^47^](#_ENREF_47) | 15 | -29.44 |
| Arkhangelskaya Oblast | Not endemic | Absent | -3 | NA | NA | -6 | High | NA | NA | -100.00 |
| Arunachal Pradesh | Unspecified | Absent | -2 | NA | NA | 2 | Low | NA | NA | 0.00 |
| Astrakhanskaya Oblast | Endemic | 50+ cases | 3 | 2007, Reported | 4 | 6 | High | NA | NA | 86.67 |
| Bashkortostan Rep. | Not endemic | Absent | -3 | NA | NA | -6 | High | NA | NA | -100.00 |
| Bassas da India | Unspecified | Absent | -2 | NA | NA | -6 | High | NA | NA | -88.89 |
| Beijing Shi | Not endemic | *Hyalomma* ticks | -2 | NA | NA | -2 | Medium | NA | NA | -44.44 |
| Belgorodskaya Oblast | Not endemic | 50+ cases | -0.5 | NA | NA | -6 | High | NA | NA | -72.22 |
| Bryanskaya Oblast | Not endemic | Absent | -3 | NA | NA | -6 | High | NA | NA | -100.00 |
| Buryatiya Rep. | Not endemic | Absent | -3 | NA | NA | -6 | High | NA | NA | -100.00 |
| Chechnya Rep. | Not endemic | 50+ cases | -0.5 | NA | NA | -6 | High | NA | NA | -72.22 |
| Chelyabinskaya Oblast | Not endemic | Absent | -3 | NA | NA | -6 | High | NA | NA | -100.00 |
| Chitinskaya Oblast | Not endemic | Absent | -3 | NA | NA | -6 | High | NA | NA | -100.00 |
| Chongqing Shi | Not endemic | *Hyalomma* ticks | -2 | NA | NA | -2 | Medium | NA | NA | -44.44 |
| Chukotskiy Okrug | Not endemic | Absent | -3 | NA | NA | -6 | High | NA | NA | -100.00 |
| Chuvashiya Rep. | Not endemic | Absent | -3 | NA | NA | -6 | High | NA | NA | -100.00 |
| Clipperton Island | Unspecified | Absent | -2 | NA | NA | -6 | High | NA | NA | -88.89 |
| Dagestan Rep. | Endemic | 50+ cases | 3 | 2001, Reported | 3 | 4 | High | NA | NA | 66.67 |
| Evenkiyskiy Okrug | Not endemic | Absent | -3 | NA | NA | -6 | High | NA | NA | -100.00 |
| Fujian Sheng | Not endemic | *Hyalomma* ticks | -2 | NA | NA | -2 | Medium | NA | NA | -44.44 |
| Gansu Sheng | Not endemic | *Hyalomma* ticks | -2 | NA | NA | -2 | Medium | NA | NA | -44.44 |
| Gaza Strip | Unspecified | Absent | -2 | NA | NA | -2 | Medium | NA | NA | -44.44 |
| Guangdong Sheng | Not endemic | *Hyalomma* ticks | -2 | NA | NA | -2 | Medium | NA | NA | -44.44 |
| Guangxi Zhuangzu Zizhiqu | Not endemic | *Hyalomma* ticks | -2 | NA | NA | -2 | Medium | NA | NA | -44.44 |
| Guizhou Sheng | Not endemic | *Hyalomma* ticks | -2 | NA | NA | -2 | Medium | NA | NA | -44.44 |
| Hainan Sheng | Not endemic | *Hyalomma* ticks | -2 | NA | NA | -2 | Medium | Human seroprevalence, 2010[^47^](#_ENREF_47) | 15 | -29.44 |
| Hebei Sheng | Not endemic | *Hyalomma* ticks | -2 | NA | NA | -2 | Medium | NA | NA | -44.44 |
| Heilongjiang Sheng | Not endemic | *Hyalomma* ticks | -2 | NA | NA | -2 | Medium | NA | NA | -44.44 |
| Henan Sheng | Not endemic | *Hyalomma* ticks | -2 | NA | NA | -2 | Medium | NA | NA | -44.44 |
| Hubei Sheng | Not endemic | *Hyalomma* ticks | -2 | NA | NA | -2 | Medium | NA | NA | -44.44 |
| Hunan Sheng | Not endemic | *Hyalomma* ticks | -2 | NA | NA | -2 | Medium | NA | NA | -44.44 |
| Ingushetiya Rep. | Endemic | 50+ cases | 3 | 2007, Reported | 3 | 4 | High | NA | NA | 66.67 |
| Irkutskaya Oblast | Not endemic | Absent | -3 | NA | NA | -6 | High | NA | NA | -100.00 |
| Isle of Man | Not endemic | Absent | -3 | NA | NA | -6 | High | NA | NA | -100.00 |
| Ivanovskaya Oblast | Not endemic | Absent | -3 | NA | NA | -6 | High | NA | NA | -100.00 |
| Jiangsu Sheng | Not endemic | *Hyalomma* ticks | -2 | NA | NA | -2 | Medium | NA | NA | -44.44 |
| Jiangxi Sheng | Not endemic | *Hyalomma* ticks | -2 | NA | NA | -2 | Medium | NA | NA | -44.44 |
| Jilin Sheng | Not endemic | *Hyalomma* ticks | -2 | NA | NA | -2 | Medium | NA | NA | -44.44 |
| Kabardino-balkariya Rep. | Not endemic | 50+ cases | -0.5 | NA | NA | -6 | High | NA | NA | -72.22 |
| Kaliningradskaya Oblast | Not endemic | Absent | -3 | NA | NA | -6 | High | NA | NA | -100.00 |
| Kalmykiya Rep. | Endemic | 50+ cases | 3 | 2006, Reported | 6 | 6 | High | NA | NA | 100.00 |
| Kaluzhskaya Oblast | Not endemic | Absent | -3 | NA | NA | -6 | High | NA | NA | -100.00 |
| Kamchatskaya Oblast | Not endemic | Absent | -3 | NA | NA | -6 | High | NA | NA | -100.00 |
| Karatchayevo-cherkesiya Rep. | Not endemic | 50+ cases | -0.5 | 2007, Reported | 4 | -6 | High | NA | NA | -16.67 |
| Karelya Rep. | Not endemic | Absent | -3 | NA | NA | -6 | High |  |  | -100.00 |
| Kashmir | Unspecified | Absent | -2 | NA | NA | 2 | Low | Human seroprevalence; Animal: camel, donkey, cattle, buffalo, horse, mule, sheep, goat[^48^](#_ENREF_48) | 15 | 15.00 |
| Kemerovskaya Oblast | Not endemic | Absent | -3 | NA | NA | -6 | High | NA | NA | -100.00 |
| Khabarovskiy Kray | Not endemic | Absent | -3 | NA | NA | -6 | High | NA | NA | -100.00 |
| Khakasiya Rep. | Not endemic | Absent | -3 | NA | NA | -6 | High | NA | NA | -100.00 |
| Khanty-mansyiskiy Okrug | Not endemic | Absent | -3 | NA | NA | -6 | High | NA | NA | -100.00 |
| Kirovskaya Oblast | Not endemic | Absent | -3 | NA | NA | -6 | High | NA | NA | -100.00 |
| Komi Rep. | Not endemic | Absent | -3 | NA | NA | -6 | High | NA | NA | -100.00 |
| Komi-permyatskiy Okrug | Not endemic | Absent | -3 | NA | NA | -6 | High | NA | NA | -100.00 |
| Koryakskiy Okrug | Not endemic | Absent | -3 | NA | NA | -6 | High | NA | NA | -100.00 |
| Kostromskaya Oblast | Not endemic | Absent | -3 | NA | NA | -6 | High | NA | NA | -100.00 |
| Krasnodarskiy Kray | Not endemic | 50+ cases | -0.5 | 2005, Reported | 6 | -6 | High | NA | NA | -0.03 |
| Kurganskaya Oblast | Not endemic | Absent | -3 | NA | NA | -6 | High | NA | NA | -100.00 |
| Kurskaya Oblast | Endemic | Absent | 0 | NA | NA | -6 | High | NA | NA | -66.67 |
| Leningradskaya Oblast | Not endemic | Absent | -3 | NA | NA | -6 | High | NA | NA | -100.00 |
| Liaoning Sheng | Not endemic | *Hyalomma* ticks | -2 | NA | NA | -2 | Medium | NA | NA | -44.44 |
| Lipetskaya Oblast | Not endemic | Absent | -3 | NA | NA | -6 | High | NA | NA | -100.00 |
| Magadanskaya Oblast | Not endemic | Absent | -3 | NA | NA | -6 | High | NA | NA | -100.00 |
| Mariy-el Rep. | Not endemic | Absent | -3 | NA | NA | -6 | High | NA | NA | -100.00 |
| Mordoviya Rep. | Not endemic | Absent | -3 | NA | NA | -6 | High | NA | NA | -100.00 |
| Moskovskaya Oblast | Not endemic | Absent | -3 | NA | NA | -6 | High | NA | NA | -100.00 |
| Moskva | Not endemic | Absent | -3 | NA | NA | -6 | High | NA | NA | -100.00 |
| Murmanskaya Oblast | Not endemic | Absent | -3 | NA | NA | -6 | High | NA | NA | -100.00 |
| Name Unknown/Kalmykia | Endemic | 50+ cases | 3 | 2006, Reported | 3 | -6 | High | NA | NA | 0.00 |
| Nei Mongol Zizhiqu | Not endemic | *Hyalomma* ticks | -2 | NA | NA | -2 | Medium | Human seroprevalence, 2010[^47^](#_ENREF_47) | 15 | -29.44 |
| Nenetskiy Okrug | Not endemic | Absent | -3 | NA | NA | -6 | High | NA | NA | -100.00 |
| Ningxia Huizu Zizhiqu | Not endemic | *Hyalomma* ticks | -2 | NA | NA | -2 | Medium | NA | NA | -44.44 |
| Nizhegorodskaya Oblast | Not endemic | Absent | -3 | NA | NA | -6 | High | NA | NA | -100.00 |
| Novgorodskaya Oblast | Not endemic | Absent | -3 | NA | NA | -6 | High | NA | NA | -100.00 |
| Novosibirskaya Oblast | Not endemic | Absent | -3 | NA | NA | -6 | High | NA | NA | -100.00 |
| Omskaya Oblast | Not endemic | Absent | -3 | NA | NA | -6 | High | NA | NA | -100.00 |
| Orenburgskaya Oblast | Not endemic | Absent | -3 | NA | NA | -6 | High | NA | NA | -100.00 |
| Orlovskaya Oblast | Not endemic | Absent | -3 | NA | NA | -6 | High | NA | NA | -100.00 |
| Penzenskaya Oblast | Not endemic | Absent | -3 | NA | NA | -6 | High | NA | NA | -100.00 |
| Permskaya Oblast | Not endemic | Absent | -3 | NA | NA | -6 | High | NA | NA | -100.00 |
| Primorskiy Kray | Not endemic | Absent | -3 | 1967, Genotype Anagnostou & Papa, 2009 | 4 | -6 | High | NA | NA | -33.33 |
| Pskovskaya Oblast | Not endemic | Absent | -3 | NA | NA | -6 | High | NA | NA | -100.00 |
| Qinghai Sheng | Not endemic | *Hyalomma* ticks | -2 | NA | NA | -2 | Medium | Human seroprevalence, 2010[^47^](#_ENREF_47) | 15 | -29.44 |
| Rostovskaya Oblast | Not endemic | 50+ cases | -0.5 | 2008, Reported | 4 | 6 | High | NA | NA | 63.33 |
| Ryazanskaya Oblast | Not endemic | Absent | -3 | NA | NA | -6 | High | NA | NA | -100.00 |
| Sakha Rep. | Not endemic | Absent | -3 | NA | NA | -6 | High | NA | NA | -100.00 |
| Sakhalinskaya Oblast | Not endemic | Absent | -3 | NA | NA | -6 | High | NA | NA | -100.00 |
| Samarskaya Oblast | Not endemic | Absent | -3 | NA | NA | -6 | High | NA | NA | -100.00 |
| Sankt-peterburg | Not endemic | Absent | -3 | NA | NA | -6 | High | NA | NA | -100.00 |
| Saratovskaya Oblast | Not endemic | 50+ cases | -0.5 | NA | NA | -6 | High | NA | NA | -72.22 |
| Severnaya Osetiya-alaniya Rep. | Not endemic | 50+ cases | -0.5 | NA | NA | -6 | High | NA | NA | -72.22 |
| Shaanxi Sheng | Not endemic | *Hyalomma* ticks | -2 | NA | NA | -2 | Medium | NA | NA | -44.44 |
| Shandong Sheng | Not endemic | *Hyalomma* ticks | -2 | NA | NA | -2 | Medium | NA | NA | -44.44 |
| Shanghai Shi | Not endemic | *Hyalomma* ticks | -2 | NA | NA | -2 | Medium | NA | NA | -44.44 |
| Shanxi Sheng | Not endemic | *Hyalomma* ticks | -2 | NA | NA | -2 | Medium | NA | NA | -44.44 |
| Sichuan Sheng | Not endemic | *Hyalomma* ticks | -2 | NA | NA | -2 | Medium | Human seroprevalence^[47](#_ENREF_47)^ | 15 | -29.44 |
| Smolenskaya Oblast | Not endemic | Absent | -3 | NA | NA | -6 | High | NA | NA | -100.00 |
| Stavropolskiy Kray | Endemic | 50+ cases | 3 | 2007, Reported | 4 | 6 | High | NA | NA | 86.67 |
| Sverdlovskaya Oblast | Not endemic | Absent | -3 | NA | NA | -6 | High | NA | NA | -100.00 |
| Taiwan Sheng | Not endemic | *Hyalomma* ticks | -2 | NA | NA | -2 | Medium | NA | NA | -44.44 |
| Tambovskaya Oblast | Not endemic | Absent | -3 | NA | NA | -6 | High | NA | NA | -100.00 |
| Tatarstan Rep. | Not endemic | Absent | -3 | NA | NA | -6 | High | NA | NA | -100.00 |
| Taymyrskiy Okrug | Not endemic | Absent | -3 | NA | NA | -6 | High | NA | NA | -100.00 |
| Tianjin Shi | Not endemic | *Hyalomma* ticks | -2 | NA | NA | -2 | Medium | NA | NA | -44.44 |
| Tibet | Not endemic | *Hyalomma* ticks | -2 | NA | NA | -2 | Medium | NA | NA | -44.44 |
| Tomskaya Oblast | Not endemic | Absent | -3 | NA | NA | -6 | High | NA | NA | -100.00 |
| Tulskaya Oblast | Not endemic | Absent | -3 | NA | NA | -6 | High | NA | NA | -100.00 |
| Tverskaya Oblast | Not endemic | Absent | -3 | NA | NA | -6 | High | NA | NA | -100.00 |
| Tyumenskaya Oblast | Not endemic | Absent | -3 | NA | NA | -6 | High | NA | NA | -100.00 |
| Tyva Rep. | Not endemic | Absent | -3 | NA | NA | -6 | High | NA | NA | -100.00 |
| Udmurtiya Rep. | Not endemic | Absent | -3 | NA | NA | -6 | High | NA | NA | -100.00 |
| Ulyanovskaya Oblast | Not endemic | Absent | -3 | NA | NA | -6 | High | NA | NA | -100.00 |
| Ustordynskiy Buryatskiy Okrug | Not endemic | Absent | -3 | NA | NA | -6 | High | NA | NA | -100.00 |
| Vladimirskaya Oblast | Not endemic | Absent | -3 | 1967, genotype[^49^](#_ENREF_49) | 4 | -6 | High | NA | NA | -33.33 |
| Volgogradskaya Oblast | Endemic | 50+ cases | 3 | 2007, Reported | 4 | 6 | High | NA | NA | 86.67 |
| Vologodskaya Oblast | Not endemic | Absent | -3 | NA | NA | -6 | High | NA | NA | -100.00 |
| Voronezhskaya Oblast | Not endemic | 50+ cases | -0.5 | NA | NA | -6 | High | NA | NA | -72.22 |
| Wake Island | Not endemic | Absent | -3 | NA | NA | -6 | High | NA | NA | -100.00 |
| Xinjiang Uygur Zizhiqu | Endemic | 5–49 cases | 2.5 | 1988, genotype[^50^](#_ENREF_50) | 2 | 2 | Medium | NA | NA | 43.33 |
| Xizang Zizhiqu | Not endemic | *Hyalomma* ticks | -2 | NA | NA | -2 | Medium | NA | NA | -44.44 |
| Yamalo-nenetskiy Okrug | Not endemic | Absent | -3 | NA | NA | -6 | High | NA | NA | -100.00 |
| Yaroslavskaya Oblast | Not endemic | Absent | -3 | NA | NA | -6 | High | NA | NA | -100.00 |
| Yevreyskaya A. Oblast | Not endemic | Absent | -3 | NA | NA | -6 | High | NA | NA | -100.00 |
| Yunnan Sheng | Unspecified | *Hyalomma* ticks | 0.5 | 1982, Reported[^51^](#_ENREF_51) | 2 | -2 | Medium | Human seroprevalence^[51](#_ENREF_51)^ | 15 | 18.33 |
| Zhejiang Sheng | Not endemic | *Hyalomma* ticks | -2 | NA | NA | -2 | Medium | NA | NA | -44.44 |

GIDEON: Global Infectious Diseases and Epidemiology Online Network; HES: healthcare expenditure score; HOS: health organisation score; LS: literature score; NA: not applicable; SIS: supplementary information score; Viro/sero: virological/serological.

**References**

1. Atkinson B, Chamberlain J, Jameson LJ et al. Identification and analysis of Crimean-Congo hemorrhagic fever virus from human sera in Tajikistan. Int J Infect Dis 2013;17:1031–7.

2. Papa A, Bino S, Llagami A et al. Crimean-Congo hemorrhagic fever in Albania, 2001. Eur J Clin Microbiol Infect Dis 2002;21:603–6.

3. Christova I, Di Caro A, Papa A et al. Crimean-Congo hemorrhagic fever, southwestern Bulgaria. Emerg Infect Dis 2009;15:983–5.

4. Gonzalez JP, Josse R, Johnson ED et al. Antibody prevalence against haemorrhagic fever viruses in randomized representative Central African populations. Res Virol 1989;140:319–31.

5. Chantal J, Dorchies P, Legueno B. A study on some zoonoses in Djibouti Republic .1. ruminants from Djibouti slaughterhouse. Rev Med Vet (Toulouse) 1994;145: 633–40.

6. Wood O, Lee V, Ash J et al. Crimean-Congo hemorrhagic fever, Thogoto, Dugbe, and Jos viruses isolated from Ixodid ticks in Ethiopia. Am J Trop Med Hyg 1978;27:600–4.

7. Zakhashvili K, Tsertsvadze N, Chikviladze T et al. Crimean-Congo hemorrhagic fever in man, Republic of Georgia, 2009. Emerg Infect Dis 2010;16:1326–8.

8. Papa A, Maltezou HC, Tsiodras S et al. A case of Crimean-Congo haemorrhagic fever in Greece, June 2008. Euro Surveill 2008;13pii=18952.

9. Antoniadis A, Casals J. Serological evidence of human infection with Congo-Crimean hemorrhagic fever virus in Greece. Am J Trop Med Hyg 1982;31:1066–7.

10. Papa A, Sidira P, Kallia S et al. Factors associated with IgG positivity to Crimean-Congo hemorrhagic fever virus in the area with the highest seroprevalence in Greece. Ticks Tick Borne Dis 2013;4:417–20.

11. Sidira P, Maltezou HC, Haidich A et al. Seroepidemiological study of Crimean-Congo hemorrhagic fever in Greece, 2009–2010. Clin Microbiol Infect 2011;18:E16–9.

12. Butenko A. Arbovirus circulation in the Republic of Guinea. Med Parazitol (Mosk) 1996;2:40–5.

13. Hornok S, Horvath G. First report of adult Hyalomma marginatum rufipes (vector of Crimean-Congo haemorrhagic fever virus) on cattle under a continental climate in Hungary. Parasit Vectors 2012;5:170.

14. Horvaith L Precipitating antibodies to Crimean haemorrhagic fever virus in human sera collected in Hungary. Acta Microbiol Acad Sci Hung 1976;23:331–5.

15. Mourya DT, Yadav PD, Shete AM et al. Detection, isolation and confirmation of Crimean-Congo hemorrhagic fever virus in human, ticks and animals in Ahmadabad, India, 2010–2011. PLoS Negl Trop Dis 2012;6:e1653.

16. Chinikar S, Shah-Hosseini N, Bouzari S et al. New circulating genomic variant of Crimean-Congo hemorrhagic fever virus in Iran. Arch Virol 2013;158:1085–8.

17. Majeed B, Dicker R, Nawar A et al. Morbidity and mortality of Crimean-Congo hemorrhagic fever in Iraq: cases reported to the National Surveillance System, 1990–2010. Trans R Soc Trop Med Hyg 2012;106:480–3.

18. Tumanova IY, Seregin SV, Vyshemirski OI et al. Genetic monitoring of the Crimean-Congo hemorrhagic fever virus in Kazakhstan and Tajikistan in 2001–2003. Mol Gen Microbiol Virol 2006;2:36–41.

19. Dunster L, Dunster M, Ofula V et al. First documentation of human Crimean-Congo hemorrhagic fever, Kenya. Emerg Infect Dis 2002;8:1005–6.

20. Risaliev D Contact of the population with disease vectors in a natural focus of Crimean hemorrhagic fever in Osh Province. Zdravookhr Kirg 1979:39–41.

21. Nabeth P, Cheikh DO, Lo B et al. Crimean-Congo hemorrhagic fever, Mauritania. Emerg Infect Dis 2004;10:2143–9.

22. Palomar AM, Portillo A, Santibanez P et al. Crimean-Congo hemorrhagic fever virus in ticks from migratory birds, Morocco. Emerg Infect Dis 2013;19:260–3.

23. Burt FJ, Swanepoel R Molecular epidemiology of African and Asian Crimean-Congo haemorrhagic fever isolates. Epidemiol Infect 2005;133:659–66.

24. Mariner J, Morrill J, Ksiazek TG Antibodies to hemorrhagic fever viruses in domestic livestock in Niger: Rift Valley Fever and Crimean-Congo hemorrhagic fever. Am J Trop Med Hyg 1995;53:217–21.

25. Sanchez AJ, Vincent MJ, Nichol ST Characterization of the glycoproteins of Crimean-Congo hemorrhagic fever virus. J Virol 2002;76:7263–75.

26. Al–Zadjali M, Al–Hashim H, Al–Ghailani M et al. A case of Crimean-Congo hemorrhagic fever in Oman. Oman Med J 2013;28:210–2.

27. Williams R, Al-Busaidy A, Mehta F et al. Crimean-Congo haemorrhagic fever: a seroepidemiological and tick survey in the Sultanate of Oman. Trop Med Int Health 2000;5:99–106.

28. Boston Children's Hospital. HealthMap. Boston, MA: Boston Children's Hospital; 2015. <http://www.healthmap.org/> [accessed: 15 May 2015].

29. Filipe A, Calisher C, Lazuick J Antibodies to Congo-Crimean haemorrhagic fever, Dhori, Thogoto and Bhanja viruses in southern Portugal. Acta Virol 1985;29:324–8.

30. Ceianu CS, Panculescu-Gatej RI, Coudrier D et al. First serologic evidence for the circulation of Crimean–Congo hemorrhagic fever virus in Romania. Vector Borne Zoonotic Dis 2012;12:718–21.

31. El-Azazy OME, Scrimgeour EM Crimean-Congo haemorrhagic fever virus infection in the Western Province of Saudi Arabia. Trans R Soc Trop Med Hyg 1997;91:275–8.

32. Tarantola A, Nabeth P, Tattevin P et al. Lookback exercise with imported Crimean–Congo hemorrhagic fever, Senegal and France. Emerg Infect Dis 2006;12:1424–6.

33. Humolli I, Dedushaj I, Zupanac TA et al. Epidemiological, serological and herd immunity of Crimean-Congo haemorrhagic fever in Kosovo. Med Arh 2010;64:91–3.

34. International Society for Infectious Diseases. ProMED mail. Brookline MA: International Society for Infectious Diseases; 2015. <http://www.promedmail.org/> [accessed 15 May 2015].

35. Elata AT, Karsany MS, Elageb RM et al. A nosocomial transmission of crimean–congo hemorrhagic fever to an attending physician in north Kordufan, Sudan. Virol J 2011;8:303.

36. Midilli K, Gargili A, Ergonul O et al. The first clinical case due to AP92 like strain of Crimean-Congo hemorrhagic fever virus and a field survey. BMC Infect Dis 2009;9:90.

37. Meissner JD, Seregin SS, Seregin SV et al. A variable region in the Crimean-Congo hemorrhagic fever virus L segment distinguishes between strains isolated from different geographic regions. J Med Virol 2006;78:223–8.

38. Smirnova S, Mamaev V, Nepesova N et al. Study of the circulation of Crimean hemorrhagic fever virus in Turkmenistan. Zh Mikrobiol Epidemiol Immunobiol 1978;1:92–7.

39. Rodhain F, Gonzalez J, Mercier E et al. Arbovirus infections and viral haemorrhagic fevers in Uganda: a serological survey in Karamoja district, 1984. Trans R Soc Trop Med Hyg 1989;83:851–4.

40. Markeshin S, Smirnova S, Evstafev I. An evaluation of the status of natural foci of Crimean-Congo hemorrhagic fever in the Crimea. Zh Mikrobiol Epidemiol Immunobiol 1991;9:47–50.

41. Schwarz TF, Nsanze H, Ameen AM. Clinical features of Crimean-Congo haemorrhagic fever in the United Arab Emirates. Infection 1997;25:364–7.

42. Rodriguez LL, Maupin GO, Ksiazek TG et al. Molecular investigation of a multisource outbreak of Crimean–Congo hemorrhagic fever in the United Arab Emirates. Am J Trop Med Hyg 1997;57:512–8.

43. Lukashev AN Evidence for recombination in Crimean-Congo hemorrhagic fever virus. J Gen Virol 2005;86:2333–8.

44. Blackburn NK, Searle L, Taylor P. Viral haemorrhagic fever antibodies in Zimbabwe schoolchildren. Trans R Soc Trop Med Hyg 1982;76:803–5.

45. Swanepoel R, Shepherd A, Leman P et al. Epidemiologic and clinical features of Crimean–Congo hemorrhagic fever in southern Africa. Am J Trop Med Hyg 1987;36:120–32.

46. Shepherd A, Swanepoel R, Shepherd S et al. Antibody to Crimean-Congo hemorrhagic fever virus in wild mammals from southern Africa. Am J Trop Med Hyg 1987;36:133–42.

47. Wu X, Na R, Wei S et al. Distribution of tick–borne diseases in China. Parasit Vectors 2013;6:119–27.

48. Rodriquez F, Padbidri V, Ghalsasi G et al. Prevalence of Crimean haemorrhagic fever–Congo virus in Jammu & Kashmir state. Indian J Med Res 1986;84:134–8.

49. Anagnostou V, Papa A. Evolution of Crimean-Congo hemorrhagic fever virus. Infect Genet Evol 2009;9:948–54.

50. Morikawa S, Qing T, Zhao XQ et al. Genetic diversity of the M RNA segment among Crimean–Congo hemorrhagic fever virus isolates in China. Virology 2002;296: 159–64.

51. Xia H, Li P, Yang J et al. Epidemiological survey of Crimean-Congo hemorrhagic fever virus in Yunnan, China, 2008. Int J Infect Dis 2011;15:e459–63.
